# Bioinformatic Analyses of Unique (Orphan) Core Genes of the Genus *Acidithiobacillus*: Functional Inferences and Use As Molecular Probes for Genomic and Metagenomic/Transcriptomic Interrogation

**DOI:** 10.3389/fmicb.2016.02035

**Published:** 2016-12-27

**Authors:** Carolina González, Marcelo Lazcano, Jorge Valdés, David S. Holmes

**Affiliations:** ^1^Center for Bioinformatics and Genome Biology, Fundación Ciencia & VidaSantiago, Chile; ^2^Facultad de Ciencias Biologicas, Universidad Andres BelloSantiago, Chile; ^3^Center for Genomics and Bioinformatics, Faculty of Sciences, Universidad MayorSantiago, Chile

**Keywords:** *Acidithiobacillus*, *Thermithiobacillus*, extreme acidophile, Orphan (ORFan) genes, horizontal gene transfer (HGT), biomining bioleaching and acid mine drainage (AMD), acid resistance, metagenome and metatranscriptome

## Abstract

Using phylogenomic and gene compositional analyses, five highly conserved gene families have been detected in the core genome of the phylogenetically coherent genus *Acidithiobacillus* of the class *Acidithiobacillia*. These core gene families are absent in the closest extant genus *Thermithiobacillus tepidarius* that subtends the *Acidithiobacillus* genus and roots the deepest in this class. The predicted proteins encoded by these core gene families are not detected by a BLAST search in the NCBI non-redundant database of more than 90 million proteins using a relaxed cut-off of 1.0e^−5^. None of the five families has a clear functional prediction. However, bioinformatic scrutiny, using pI prediction, motif/domain searches, cellular location predictions, genomic context analyses, and chromosome topology studies together with previously published transcriptomic and proteomic data, suggests that some may have functions associated with membrane remodeling during cell division perhaps in response to pH stress. Despite the high level of amino acid sequence conservation within each family, there is sufficient nucleotide variation of the respective genes to permit the use of the DNA sequences to distinguish different species of *Acidithiobacillus*, making them useful additions to the armamentarium of tools for phylogenetic analysis. Since the protein families are unique to the *Acidithiobacillus* genus, they can also be leveraged as probes to detect the genus in environmental metagenomes and metatranscriptomes, including industrial biomining operations, and acid mine drainage (AMD).

## Introduction

The power of comparative genomics to enlighten evolutionary processes through hypotheses has emerged based on the enormous availability of complete and partial genome sequences from both early and late branching lineages at different taxonomic levels (MacLean et al., 2009). At present, we are able to exploit the powerful analytical methods of molecular evolution and population genomics to determine the relative contribution of the different evolutionary forces that shape genome organization, structure, and diversity. These methods also offer an exceptional opportunity to explore the genetic and genomic determinants of lifestyle diversity in bacteria, especially for polyextremophiles including those that thrive in extremely acidic environments and for which there are genome sequences available (Cárdenas et al., 2016a,b).

The genus *Acidithiobacillus* (termed *Acidithiobacilli*) consists of seven recognized species; *Acidithiobacillus ferrooxidans, A. ferridurans, A. ferrivorans, A. ferriphilus, A. thiooxidans, A. caldus and A. albertensis* (reviewed in Nuñez et al., 2016). The *Acidithiobacilli* together with *Thermithiobacillus tepidarius* constitute the class *Acidithiobacillia* (Williams and Kelly, 2013; Hudson et al., 2014).

The *Acidithiobacilli* have been found principally in industrial biomining and coal processing operations, the deep subsurface of the Spanish pyritic belt and in natural and man-made acid drainages including acid mine drainage (AMD; Méndez-García et al., 2015; Hedrich, 2016). All are extreme acidophiles with a pH optima for growth of 3.5 or less (Barrie Johnson and Quatrini, 2016). In contrast, *T. tepidarius* is a neutrophile that was recovered from a terrestrial thermal spring (Wood and Kelly, 1985). All the other extant bacterial lineages phylogenetically closely related to *T. tepidarius* are also neutrophiles, making it likely that the last common ancestor before the split between *T. tepidarius* and the *Acidithiobacilli* was also a neutrophile. This raises questions about the origin and evolution of genes and mechanisms that allowed the transition to be made from a neutral pH environment to an extremely acidic environment eventually giving rise to the *Acidithiobacilli*.

Mechanisms used by extreme acidophiles to mitigate the effect of low pH have been extensively investigated (Baker-Austin and Dopson, 2007). However, there are no studies that use comparative genomics to discover new genetic determinants of pH homeostasis in the *Acidithiobacilli*, although one study used multiple strains of *A. thiooxidans* to confirm known acid resistant determinants and assign them to the core or accessory genome (Zhang et al., 2016).

The study of unique gene families from extreme acidophile representatives could provide evidence about events of protein lineage specification involving many structural rearrangements needed to survive under extreme life conditions. Gene tree analyses suggest recent, lineage-specific expansion, and diversification among homologs encoding yet unknown functions for pathway and processes that might be unique requirements in *Acidithiobacilli*. Their analysis could help close gaps in our understanding of genetic and metabolic requirements that support extremophile lifestyles and they could also provide novel candidate sequences for prospecting for new DNA-based screenings and other production avenues (Sabir et al., 2016).

In the present study, we perform an extensive bioinformatic characterization of five protein families taxonomically restricted to the *Acidithiobacilli*. Analyses of their fundamental properties combined with comparative genomics and phylogenomics suggest potential functional roles and allow evolutionary models to be built. The sequences of the five families are also exploited as molecular probes for phylogenetic scrutiny and interrogation of metagenomes and metatranscriptomes including AMD and biomining operations.

## Materials and methods

### Genomes used

Table 1 provides information about the genomes.

**Table 1:** **Genomes used in this study**.

**Microorganism**	**Genome size (Mbp)**	**Predicted protein coding sequences**	**Genome G+C (%)**	**Genome accession number (NCBI)**	**References**
*Acidithiobacillus ferrooxidans* ATCC 23270^T^	2.98	3147	58.8	CP001219	Valdés et al., 2008
*Acidithiobacillus ferrooxidans* ATCC 53993	2.88	2826	58.9	CP001132	Lucas et al., 2008, Unpublished
*Acidithiobacillus ferrivorans* SS3^T^	3.2	3093	56.6	CP002985	Liljeqvist et al., 2011
*Acidithiobacillus ferrivorans* CF27	3.42	3854	56.4	CCCS020000000	Talla et al., 2014
*Acidithiobacillus thiooxidans* A01	3.82	3826	53.1	AZMO00000000	Yin et al., 2014
*Acidithiobacillus thiooxidans* ATCC 19377^T^	3.01	3041	53.1	AFOH00000000	Valdés et al., 2011
*Acidithiobacillus thiooxidans* Licanantay	3.93	4191	52.8	JMEB00000000	Travisany et al., 2014
*Acidithiobacillus caldus* ATCC 51756^T^	2.77	2681 (0.21)^P^	61.4	CP005986-CP005989	Valdes et al., 2009
*Acidithiobacillus caldus* SM-1	2.93	2881 (0.31)^P^	61.3	CP002573-CP002577	You et al., 2011
*Thermithiobacillus tepidarius* DSM 3134^T^	2.96	2750	66.8	AUIS00000000	Kelly and Wood, 2000
*Acidithiobacillus ferrooxidans* strain BY0502	2.97	2822	56.8	LVXZ00000000	Zhou, 2016, Unpublished
*Acidithiobacillus ferrooxidans* strain DLC-5	4.23	5600	57.6	JNNH00000000^*^	Chen et al., 2015
*Acidithiobacillus ferrooxidans* strain YQH-1	3.11	2949	58.6	LJBT00000000	Yan et al., 2015
*Acidithiobacillus ferrooxidans* strain Hel18	3.11	2939	58.6	LQRJ00000000	Schopf, 2016, Unpublished
*Acidithiobacillus caldus* strain MTH-04	2.87	2646	61.4	LXQG00000000	Mi et al., 2006, Unpublished
*Acidithiobacillus thiooxidans* DMC	3.85	3768	53.1	LWSB00000000	Zhang et al., 2016

T, denotes type strain;

P, *denotes plasmid information*.

* *Denotes JGI accession number*.

### Pipeline used for compiling and analyzing the data set

Predicted protein sequences corresponding to all *Acidithiobacilli* proteomes were sorted using an all-vs.-all BLASTP script based on Best Bidirectional BLAST Hit (BBBH; Altschul et al., 1997) with an *E*-value of 1e-5. Protein families were constructed based on 50% of identity and 50% of coverage in the alignments (Altschul et al., 1997), assigning each protein to one protein family. The families with predicted proteins shared by all strains were selected and denominated the core-genome (Williams and Kelly, 2013; Hudson et al., 2014). The *Acidithiobacillus* core-genome was compared using BLASTP version 2.2.26 (Altschul et al., 1997) against NCBI non-redundant (NR) database in August of 2015, using a minimal *E*-value of 1e-5. Core families with exclusive similarity with *Acidithiobacillus* members, and not associated with any other microorganism, were selected and denominated unique (orphan) core genes. The selected unique protein families were checked manually using BLASTP, Psi-BLAST (Altschul et al., 1997) and HMMer version 3.0 (Eddy, 1998) against NR database with an *E*-value of 1e-4 to confirm their exclusive association with the *Acidithiobacillus* genus. The locus tags of the respective genes are provided in Table 2.

**Table 2:** **Predicted properties of the proteins of families I–V**.

	**Microorganism**	**Locus tag or contig**	**pI**	**Size (aa)**	**TM regions**	**Signal peptide**	**Subcellular location**	**Lipoprotein signature**
Family I	*A. ferrooxidans* ATCC 23270	AFE_0294	8.06	250	5	–	IM	–
	*A. ferrooxidans* ATCC 53993	Lferr_0470	8.06	251	5	–	IM	–
	*A. ferrivorans* SS3	Acife_2737	9.47	259	5	–	IM	–
	*A. ferrivorans* CF27	CDQ10770.1	9.26	259	5	–	IM	–
	*A. thiooxidans* ATCC 19377	AFOH01000117	8.21	261	5	–	IM	–
	*A. thiooxidans* A01	AZMO01000067	8.06	263	5	–	IM	–
	*A. thiooxidans* Licanantay	JMEB01000250	8.21	261	5	–	IM	–
	*A. caldus* SM-1	Atc_0578	9.25	257	5	–	IM	–
	*A. caldus* ATCC 51756	Acaty_c0588	8.85	249	5	–	IM	–
Family II	*A. ferrooxidans* ATCC 23270	AFE_2894	9.52	103	1	–	IM/P/C	–
	*A. ferrooxidans* ATCC 53993	Lferr_2514	9.52	103	1	–	IM	–
	*A. ferrivorans* SS3	Acife_0262	10.26	103	1	–	IM/P/C	–
	*A. ferrivorans* CF27	CDQ10832.1	9.98	103	1	–	IM/P/C	–
	*A. thiooxidans* ATCC 19377	AFOH01000056	10.94	103	1	–	IM/P/C	–
	*A. thiooxidans* A01	AZMO01000007	10.63	103	1	–	IM/P/C	–
	*A. thiooxidans* Licanantay	JMEB01000152	10.90	103	1	–	IM/P/C	–
	*A. caldus* SM-1	Atc_0665	10.37	103	1	–	IM/P/C	–
	*A. caldus* ATCC 51756	Acaty_c0696	9.97	91	1	–	IM/P/C	–
Family III	*A. ferrooxidans* ATCC 23270	AFE_2918	6.82	128	1	Yes	P	Yes
	*A. ferrooxidans* ATCC 53993	Lferr_2533	6.82	128	1	Yes	P	Yes
	*A. ferrivorans* SS3	Acife_0237	8.79	128	1	Yes	P/C	Yes
	*A. ferrivorans* CF27	CDQ10857.1	7.88	128	1	Yes	P	Yes
	*A. thiooxidans* ATCC 19377	AFOH01000056	8.76	128	1	Yes	P	Yes
	*A. thiooxidans* A01	AZMO01000007	8.07	128	1	Yes	P	Yes
	*A. thiooxidans* Licanantay	JMEB01000332	8.76	128	1	Yes	P	Yes
	*A. caldus* SM-1	Atc_2682	8.58	129	1	Yes	P/C	Yes
	*A. caldus* ATCC 51756	Acaty_c2529	8.59	129	1	Yes	P/C	Yes
Family IV	*A. ferrooxidans* ATCC 23270	AFE_3261	6.33	172	–	Yes	P/IM	Yes
	*A. ferrooxidans* ATCC 53993	Lferr_2861	6.48	172	–	Yes	P	Yes
	*A. ferrivorans* SS3	Acife_0197	8.80	170	–	Yes	P/E	Yes
	*A. ferrivorans* CF27	CDQ11656.1	8.80	170	–	Yes	P/E	Yes
	*A. thiooxidans* ATCC 19377	AFOH01000137	6.33	172	–	Yes	P	Yes
	*A. thiooxidans* A01	AZMO01000008	8.21	171	–	Yes	P	Yes
	*A. thiooxidans* Licanantay	JMEB01000258	8.22	171	–	Yes	P	Yes
	*A. caldus* SM-1	Atc_0064	8.80	170	–	Yes	P/IM	Yes
	*A. caldus* ATCC 51756	Acaty_c0059	8.80	170	–	Yes	P	Yes
Family V	*A. ferrooxidans* ATCC 23270	AFE_2816	9.30	146	1	–	P/IM	–
	*A. ferrooxidans* ATCC 53993	Lferr_2439	9.31	146	1	–	P/IM	–
	*A. ferrivorans* SS3	Acife_0333	9.75	145	1	–	P	–
	*A. ferrivorans* CF27	CDQ09308.1	9.70	145	1	–	P	–
	*A. thiooxidans* ATCC 19377	AFOH01000029	9.52	86	1	–	C/P	–
	*A. thiooxidans* A01	AZMO01000004	9.56	119	1	–	P	–
	*A. thiooxidans* Licanantay	JMEB01000081	9.40	119	1	Yes	P	–
	*A. caldus* SM-1	Atc_0233	9.21	128	1	–	P	–
	*A. caldus* ATCC 51756	Acaty_c0260	9.21	128	1	–	P	–

*IM, inner membrane; C, cytoplasm; P, periplasm*.

### Genomic contexts of unique core genes

Collinear blocks between the genomes and conservation of gene neighbors were determined by MAUVE (Darling et al., 2010), RAST (Aziz et al., 2008; Overbeek et al., 2014; Markowitz et al., 2014a) and IMG-JGI (Markowitz et al., 2014b; Dhillon et al., 2015). Genomic contexts were visualized using Artemis of Sanger (Brettin et al., 2015).

### Evaluation of HGT

IslandViewer (Rutherford et al., 2000) was used to predict genomic islands.

### Annotation of unique core genes (families I–V)

Protein coding sequences were annotated using an integrated pipeline consisting of BLASTP (Altschul et al., 1997) searches against NR database of NCBI with an *E*-value cutoff of 1e-3, Pfam (Punta et al., 2012), TigrFAM (Consortium, 2014), and Uniprot (Hofmann and Stoffel, 1993) database comparisons. Transmembrane regions in protein sequences were predicted with TMHMM (Haft et al., 2003) and TMPRED (Krogh et al., 2001). Computation of isoelectric point and molecular weight were made with ExPASy web tool (Bjellqvist et al., 1993; Nakai and Horton, 1999; Gasteiger et al., 2005).

### Estimation of mutation rates

Synonymous and non-synonymous substitution rates were calculated as follows: amino acid alignments of unique (orphan) core genes were constructed using MUSCLE (Edgar, 2004), and used as input for PAL2NAL (Suyama et al., 2006) with the nucleotide sequences to create the codon alignments of gene core families. The ratio of non-synonymous (K_a_) to synonymous (K_s_) nucleotide substitution rates (K_a_/K_s_ ratios) were calculated using SeqinR package of R project (Charif and Lobry, 2007). Mean K_a_/K_s_ ratios were assigned for individual unique (orphan) core genes (families I–V) by averaging all pairwise ratios within each family.

### Signal peptide and subcellular location predictions

A combination of computational prediction tools PSORTb (Nakai and Horton, 1999; Yu et al., 2010), CELLO (Yu et al., 2006) and ProtCompB^1^ (Yu et al., 2004) were used to perform whole genome analysis of unique core protein subcellular localization via the Sec Mechanism and Tat signal prediction (Natale et al., 2008; Bagos et al., 2010). The results derived from three prediction algorithms tools were combined according to majority to obtain a more accurate protein subcellular localization prediction.

### Lipoproteins signal prediction

Prediction of lipoproteins signals was made with LipoP Server (Juncker et al., 2003).

### Phylogenetic analyses

16S rRNA sequences from *Acidithiobacillus* genomes were identified by BLASTN-based script using an *E*-value threshold of 1e-5 and the databases GREENGENES (DeSantis et al., 2006), RDP (Cole et al., 2009) and SILVA (Pruesse et al., 2007) and were aligned using MAFFT (Katoh et al., 2002, 2005) alignment tool with L-INS strategy. Phylogenetic trees were constructed with MrBayes (Huelsenbeck and Ronquist, 2001; Ronquist and Huelsenbeck, 2003) and PHYML (Guindon et al., 2010), using the substitution model predicted for jModelTest2 (Guindon and Gascuel, 2003; Darriba et al., 2012).

### Mapping of genes for families I–V onto circular genomes

The genes encoding families I–V were mapped onto the genomes *A. ferrooxidans* ATCC 23270, *A. ferrivorans* SS3, *A. caldus* ATCC 51756, and *A. caldus* SM-1 using DNAplotter (Carver et al., 2009). The origin of replication (Ori) of each genome was predicted between *dnaN* and *dnaA* as previously described (Valdés et al., 2008) and was used as the zero coordinate to orient the genome maps.

### Metagenomic analysis

Metagenomic and metatranscriptomic sequences were downloaded from NCBI, JGI (Nordberg et al., 2014), and MG-RAST (Meyer et al., 2008; additional information can be found in **Table 4**) and were interrogated by BLASTX (Altschul et al., 1997) against the five core protein families with an *E*-value cut-off of 1e-5. The percent identity and coverage of sequences were analyzed for each alignment.

## Results and discussion

### Pipeline for discovery of protein families unique to the core genome of the genus *Acidithiobacillus*

**Figure 1:**
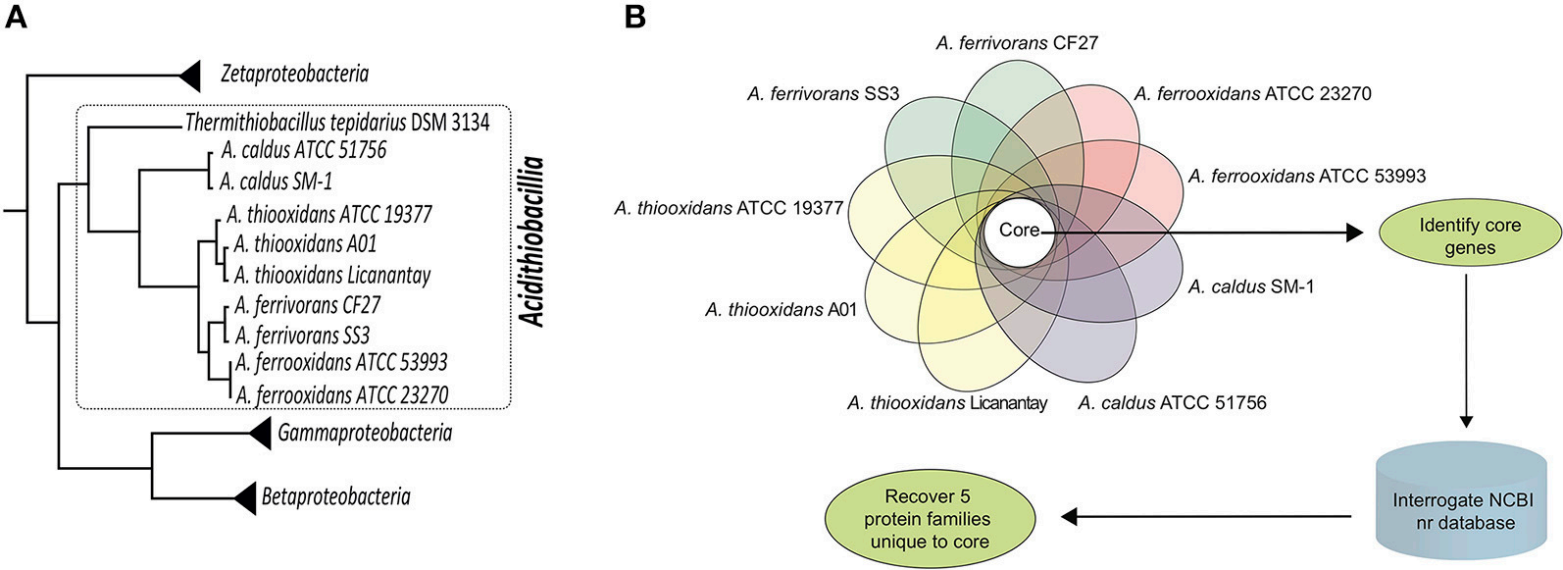
**Work Flow. (A)** Phylogenetic tree of the class *Acidithiobacillia* (within the dotted line) showing the clustering of the acidophilic *Acidithiobacillus* genus (*Acidithiobacilli*) subtended by the neutrophilic *Thermithiobacillus tepidarius*. The tree is based on genome-scale maximum-likelihood analysis of 98 universal protein families (housekeeping) conserved in *Zeta*-, *Gamma*-, *Betaproteobacteria*, and *Acidithiobacillia* class according to references Williams and Kelly (2013) and Hudson et al. (2014). **(B)** Pipeline for the identification and recovery of five protein families (termed I-V) unique to the genus *Acidithiobacillus*.

Figure 1 summarizes the bioinformatics pipeline used to recover five families of proteins and their corresponding genes that are taxonomically restricted to the genus *Acidithiobacillus*. Using a relaxed cutoff (1e-5) in a BLAST search, they were not detected in the NCBI nr database of more than 90 million proteins that includes the predicted proteins of *Thermithiobacillus tepidarius*, the nearest extant relative of the *Acidithiobacilli*.

### Integrative bioinformatics approaches can suggest functions for the unique *Acidithiobacillus* gene families I–V

Since *Acidithiobacilli*-specific protein families have almost no similarity with known proteins for other non-*Acidithiobacilli* representatives, we used a collection of bioinformatics resources in order to gain insights into potential protein functions based on hydrophobicity profiles, secondary structure predictions, predicted protein cell localizations and the comparison of consensus and profile sequences to pattern and domain databases (see Section Materials and Methods). Protein function predictions of the five *Acidithiobacilli*-specific protein families were examined using an analysis of their genomic contexts. Their differential expression was linked to previously published proteomic data derived from cells subjected to changes of pH, which is known to be a major selective pressure for members of the *Acidithiobacillus* genus (Baker-Austin and Dopson, 2007; see Table 3).

**Table 3:** **Gene expression evidence**.

	**Microorganism**	**Locus tag or contig**	**Gene expressed^a^**	**Protein abundance with pH change^b^**		**Meta-transcriptomic evidence^c^**
Family I	*A. ferrooxidans* ATCC 23270	AFE_0294	ND	ND	Family I	AFE sp. Yes
	*A. ferrooxidans* ATCC 53993	Lferr_0470	ND	ND		
	*A. ferrivorans* SS3	Acife_2737	Yes	ND		
	*A. ferrivorans* CF27	CDQ10770.1	ND	ND		AFV sp. Yes
	*A. thiooxidans* ATCC 19377	AFOH01000117	ND	ND		
	*A. thiooxidans* A01	AZMO01000067	ND	ND		
	*A. thiooxidans* Licanantay	JMEB01000250	ND	ND		ATHIO sp. Yes
	*A. caldus* SM-1	Atc_0578	ND	ND		
	*A. caldus* ATCC 51756	Acaty_c0588	Yes	Up at pH 1		
Family II	*A. ferrooxidans* ATCC 23270	AFE_2894	ND	ND	Family II	AFE sp. Yes
	*A. ferrooxidans* ATCC 53993	Lferr_2514	ND	ND		
	*A. ferrivorans* SS3	Acife_0262	Yes	ND		
	*A. ferrivorans* CF27	CDQ10832.1	ND	ND		AFV sp. Yes
	*A. thiooxidans* ATCC 19377	AFOH01000056	ND	ND		
	*A. thiooxidans* A01	AZMO01000007	ND	ND		
	*A. thiooxidans* Licanantay	JMEB01000152	ND	ND		ATHIO sp. Yes
	*A. caldus* SM-1	Atc_0665	ND	ND		
	*A. caldus* ATCC 51756	Acaty_c0696	Yes	No change		
Family III	*A. ferrooxidans* ATCC 23270	AFE_2918	Yes	ND	Family III	AFE sp. Yes
	*A. ferrooxidans* ATCC 53993	Lferr_2533	ND	ND		
	*A. ferrivorans* SS3	Acife_0237	Yes	ND		
	*A. ferrivorans* CF27	CDQ10857.1	ND	ND		AFV sp. Yes
	*A. thiooxidans* ATCC 19377	AFOH01000056	ND	ND		
	*A. thiooxidans* A01	AZMO01000007	ND	ND		
	*A. thiooxidans* Licanantay	JMEB01000332	ND	ND		ATHIO sp. Yes
	*A. caldus* SM-1	Atc_2682	ND	ND		
	*A. caldus* ATCC 51756	Acaty_c2529	Yes	Up at pH 1		
Family IV	*A. ferrooxidans* ATCC 23270	AFE_3261	ND	ND	Family IV	AFE sp. Yes
	*A. ferrooxidans* ATCC 53993	Lferr_2861	ND	ND		
	*A. ferrivorans* SS3	Acife_0197	Yes	ND		
	*A. ferrivorans* CF27	CDQ11656.1	ND	ND		AFV sp. Yes
	*A. thiooxidans* ATCC 19377	AFOH01000137	ND	ND		
	*A. thiooxidans* A01	AZMO01000008	ND	ND		
	*A. thiooxidans* Licanantay	JMEB01000258	ND	ND		ATHIO sp. Yes
	*A. caldus* SM-1	Atc_0064	ND	ND		
	*A. caldus* ATCC 51756	Acaty_c0059	Yes	Up at pH 1		
Family V	*A. ferrooxidans* ATCC 23270	AFE_2816	ND	ND	Family V	AFE sp. Yes
	*A. ferrooxidans* ATCC 53993	Lferr_2439	ND	ND		
	*A. ferrivorans* SS3	Acife_0333	Yes	ND		
	*A. ferrivorans* CF27	CDQ09308.1	ND	ND		AFV sp. Yes
	*A. thiooxidans* ATCC 19377	AFOH01000029	ND	ND		
	*A. thiooxidans* A01	AZMO01000004	ND	ND		
	*A. thiooxidans* Licanantay	JMEB01000081	ND	ND		ATHIO sp. Yes
	*A. caldus* SM-1	Atc_0233	ND	ND		
	*A. caldus* ATCC 51756	Acaty_c0260	Yes	Up at pH 4		

*Expression of members of the five orphan families in different environmental conditions. Locus tags for the five families are provided*.

a *Gene expression for families I–V was extracted from Christel et al. (2016a,b) and Osorio et al. (2013)*.

b *Information regarding protein abundance levels when A. caldus was subjected to growth at pH 1, 2, or 4 was taken from Mangold et al. (2013). Abundance of proteins is expressed as “up in low pH” or “up in high pH” relative to protein levels found at pH 2 (Mangold et al., 2013). Note that the gene accession numbers in Mangold et al. (2013) have been replaced recently by the locus tags provided in this Table*.

c *RNA transcript expression as determined by examination of published metatranscriptomics data (Chen et al., 2015) using the families I–V as probes (see Table 4 for details)*.

*AFE, Acidithiobacillus ferrooxidans; AFV, Acidithiobacillus ferrivorans; ATHIO, Acidithiobacillus thiooxidans; ND, Not detected*.

**Figure 2:**
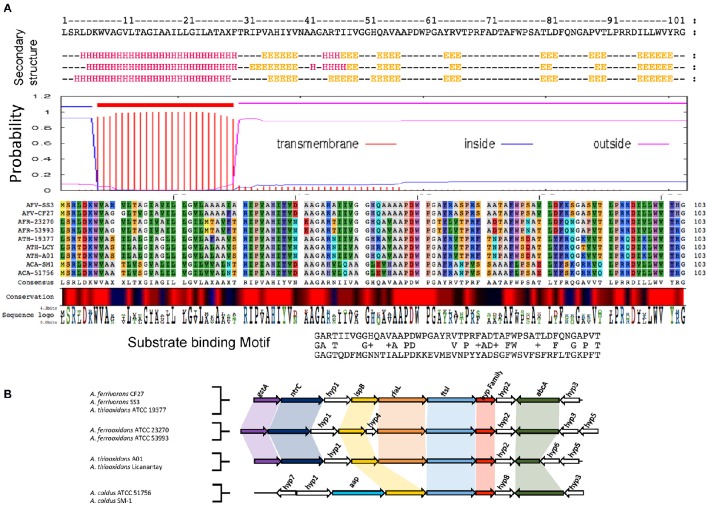
**Example of functional prediction based on multiple bioinformatics and genome-based evidence for members of family II. (A)** Bioinformatics analysis of members of family II based on secondary structure prediction, hydrophobicity profiles and transmembrane segments prediction, multiple alignments and conservation profiles for the generation of consensus and profile sequences and their comparison to specific substrate binding protein profiles found in public databases. **(B)** Genomic context analysis of members of family II including functional annotations of the closest neighborhood genes for functional association. gstA, Glutathione S-transferase; ntrC, Nitrogen assimilation regulatory protein; ispB, Octaprenyl-diphosphate synthase; rfaL, O-antigen ligase; ftsI, Cell division protein; Hyp family II, Hypothetical protein; abcA, ABC transporter A family; app, Amino acid permease; Hyp (1–8), Hypothetical protein (1–8). Table 2 provides a complete overview of the predicted properties from amino acid sequences for member of the five families.

Figure 2 provides an example of the predicted protein properties deduced with bioinformatics tools and comparative genomic analysis for members of family II. Additional information for all five families I–V can be found in Supplemental Files 1, 2. *In silico* predictions demonstrate the power of integrative genomics approaches to gain insights into gene function. A significant prediction was made for an integral membrane segment with a moderate conservation profile within the family II. From the non-membrane associated portion of the protein, profile sequences were generated that have similarity to a pattern present in periplasmic binding proteins (Dwyer and Hellinga, 2004) and also solute carrier organic anion transporter family member 4A1 (Pizzagalli et al., 2003).

Comparative genome organization data demonstrated that there is conservation of gene neighborhood profiles that include genes predicted for cell division, surface proteins and ABC transport systems (Figure 2 and Supplemental File 3). Table 2 shows a detailed overview of the predicted properties based on amino acid sequences for families I–V.

### Gene expression of families I–V

Information regarding the expression of the genes encoding the five families was extracted from the literature and is presented in Table 3. RNA transcript analysis indicates that all five family genes are expressed in *A. ferrivorans* SS3 in two different conditions: continuous culture at 20°C (Christel et al., 2016a) and at 8°C (Christel et al., 2016b), adjusted to pH 2.5 with sulfuric acid plus trace elements. A proteomic study of *A. ferrooxidans* ATCC 23270 on elemental sulfur as electron donor under aerobic and anaerobic conditions (Osorio et al., 2013) showed that family III was expressed in this strain. A proteomic study of *A. caldus* ATCC 51756 using cells grown at pH 2.5 (optimum growth pH) vs. pH 1 and 4, demonstrated up-regulation of core families I, III, and IV when cells were shifted from pH 2.5 to 1 and that family V was upregulated when cells were shifted from pH 2.5 to 4 (Table 3; Mangold et al., 2013). These data show that the genes for the five families (i) are expressed and thus are unlikely to be mis-annotated open reading frames with no coding capacity and (ii) provide evidence that families I, III, IV, and V could be involved in responses to acid stress at least in *A. caldus*. It remains to be determined if changes in RNA levels are associated with these genes in the other *Acidithiobacilli*.

In addition, RNA transcripts in metatranscriptomes of the Dabaoshan and Yunfu Pond mines in China (Chen et al., 2015) were detected that exhibited sequences similar to families I–V (Table 3, right hand side) from *A. ferrooxidans, A. ferrivorans*, and *A. thiooxidans*, although no strain specificity could be determined. This supports the idea that the five families are bona fide genes.

### Insights into protein functions

**Figure 3:**
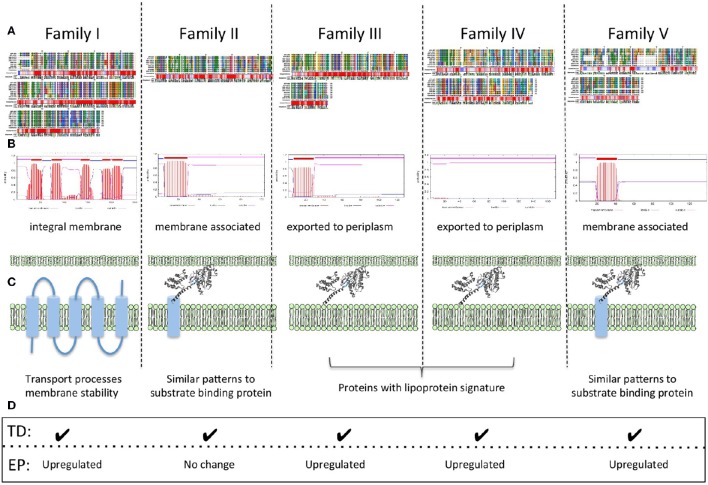
**Schematic summary of functional associations found in families I–V. (A)** Multiple alignments, conservation profiles and consensus sequences. **(B)** Transmembrane topology predictions. **(C)** Predicted protein localization and deduced general functions. **(D)** Expression data TD: RNA transcript detected; EP: protein expression profile.

In order to make a comprehensive summary of the potential gene function inferred from all the evidence presented, a schematic summary is presented in Figure 3. Families I, II, and V, have predicted transmembrane segments that, in conjunction with protein sorting signal identification, provide preliminary information about their cellular location. Profile and consensus sequences comparisons against public databases only provided information about family II. Family II sequences have motifs similar to those of periplasmic binding proteins, usually associated with ABC transport for the substrate specific incorporation of nutrients and scarce molecules or beneficial solutes under extreme environmental conditions (Cuneo et al., 2008). We suggest that members of family II could be distant relatives of periplasmic binding proteins whose specific substrate(s) and functional role remains to be investigated.

Families III and IV have predicted protein localizations associated with inner membrane and periplasmic spaces and their strong lipoprotein signatures, in addition to genomic context information, provide clues for their potential role in key physiological processes, such as lipid metabolism. We hypothesize a potential connection between membrane associated lipoproteins, lipid metabolism and membrane stability as a requirement for low pH lifestyle (Baker-Austin and Dopson, 2007; Liljeqvist et al., 2015).

Predicted protein properties of all families I–V, suggest a general involvement in functions associated with membrane processes perhaps involving roles in membrane stability, transport processes, and/or the generation of molecular components to allow the synthesis and incorporation of hydrophobic molecules into the membrane increasing its stability in low pH.

### Chromosome architecture is consistent with functional inferences (involvement in cell envelope remodeling during cell division)

It has been observed in many bacteria that the gene order relative to OriC is highly conserved along the chromosomal replicores (Sobetzko et al., 2012). Also, essential and highly expressed genes tend to be encoded close to oriC (Rocha, 2004). This heightened activity can be attributed to gene dosage effects during chromosome replication especially in rapidly dividing cells, but underlying physical properties of the circular chromosome, including an inferred gradient of DNA superhelical density from the origin to the terminus, are also known to be involved in influencing gene expression (Sobetzko et al., 2012).

**Figure 4:**
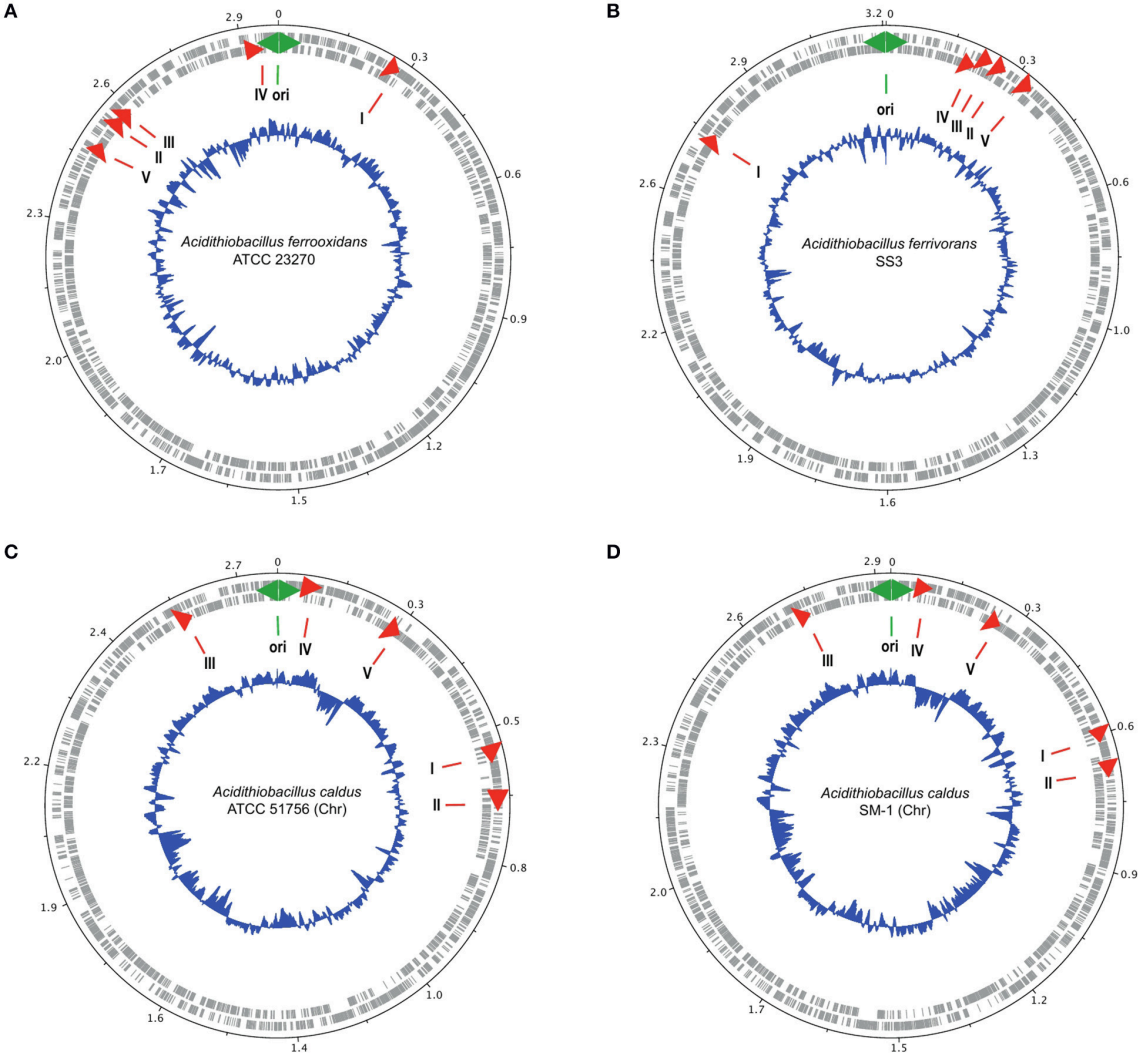
**Location of the genes encoding families I–V (red arrows) in the genomes of (A)** *A. ferrooxidans* ATCC 23270, **(B)** *A. ferrivorans* SS3, **(C)** *A. caldus* ATCC 51756, and **(D)** *A. caldus* SM-1. The outer two circles show the genes on both strands of DNA of the chromosome. The inner blue circle indicates the G+C content. The green two-headed arrow indicates the predicted origin of replication of the chromosome. The red arrows indicate the position of the families I–V genes.

In particular, it has been observed that several genes involved in acid stress, including envelope remodeling, are located close to oriC in the gammaproteobacterium *Dickeya dadantii* (Jiang et al., 2015). Given the possibility that genes of families I–V could be involved in acid stress response and that this response might be associated with chromosome topology, we determine their chromosomal locations on the closed circular chromosomes of *A. ferrooxidans* ATCC 23270^T^, *A. ferrivorans* SS3^T^, *A. caldus* ATCC 51756^T^, and *A. caldus* SM-1 using DNAplotter (Carver et al., 2009; Figure 4). In all these chromosomes, the five family genes exhibit a tendency to be located nearer Ori rather than the terminus, especially in the cases of *A. ferrooxidans* and *A. ferrivorans*. In the latter two chromosomes, the gene order relative to Ori is conserved but is inverted, perhaps due to inter-replicore translocation that is known to be common around Ori in other microorganisms (Eisen et al., 2000; Khedkar and Seshasayee, 2016). Three of the families have genes predicted to DNA handling functions in their gene neighborhoods ordered in tightly clustered associations that could be operons; for example, *rmuC* (DNA recombination) near family IV, and *dnaB* and *radA* (DNA helicase and DNA repair, respectively) near family V. These genes are usually associated with DNA replication and cell division (Figure 4). The juxtaposition of *ftsL*, an essential cell division protein (Guzman et al., 1992), to the gene encoding family II and its closeness to the family III gene (Figure 4) strongly suggests that family II and III are involved in cell division perhaps through cell envelope remodeling. Their proximity to Ori could enhance the ability of the *Acidithiobacilli* to respond to changes in environmental acidity at early stages of cell division. Such changes might be more difficult to accomplish during later stages of cell division or at the resting stage.

### Families I–V are protein coding genes

Taxonomically restricted genes, such as families I–V, are referred to as orphans genes or ORFans (orphan open reading frames; Fischer and Eisenberg, 1999; Pedroso et al., 2008; Tautz and Domazet-Loso, 2011). ORFans can be artifacts of annotation, non-coding RNA genes or protein encoding genes (Prabh and Rodelsperger, 2016). In the case of families I–V, there is evidence that those from *A. caldus* encode proteins and that families I–V from *A. ferrooxidans, A. ferrivorans*, and *A. thiooxidans* express RNA (Table 3). Given the highly conserved sequences similarity between the respective families from the different *Acidithiobacillus* species, it is reasonable to suggest that all are protein coding genes, as observed for the *A. caldus* families and are not “merely” RNA genes. However, in order to provide additional evidence for protein coding capacity, selection pressure was measured as the ratio of the synonymous and non-synonymous rates of amino acid substitution (dN/dS), also called omega (ω) for all families. The omega values for families I–V are 0.07, 0.05, 0.03, 0.05, and 0.08 respectively. An ω <1 can be interpreted as evidence for negative selection and most likely such a sequence would correspond to a protein encoding gene (Prabh and Rodelsperger, 2016). The omega values are considerably <1 for all five families providing compelling evidence that they are protein-encoding genes.

### Origin of families I–V

The genes encoding families I–V are not found in *T. tepidarius* that subtends the genus *Acidithiobacillus* and shares the last common ancestor with it, nor are they found in any other organism that has sequence information in the NCBI nr database. So questions arise as to the origin and evolution of the five families.

We propose three main hypotheses.

- The genes arose *de novo* in the *Acidithiobacillus* genus, after its split with *T. tepidarius* perhaps by gene duplication and divergence (Long et al., 2003; Tautz and Domazet-Loso, 2011; Klasberg et al., 2016). If this happened, then the duplication events occurred so long ago and/or involved such fast divergence that sequence similarities to the original genes have been blurred by subsequent evolutionary events.
- The genes entered the last common ancestor of the *Acidithiobacillus* genus by horizontal gene transfer (HGT). IslandViewer (Rutherford et al., 2000) was employed to search for evidence of HGT with no positive results. Also, the annotated gene neighborhoods of families I–V were searched by hand for evidence of signatures of HGT such as transposases (Riadi et al., 2012; Acuña et al., 2013), integrases, conjugative and viral functions, and tRNAs but only one transposase was detected in the vicinity of family IV, (Supplemental File 3). Although little evidence of HGT could be found, it can be argued that it occurred so long ago that its molecular signatures have been lost. If HGT occurred, who were the donor organisms? There is no obvious donor lineage represented in the NCBI nr database, but other organisms could remain to be discovered whose study could help shed light on the evolutionary history of the genes of families I–V genes. The increasing metagenomic sequencing efforts offer the best opportunities for discovering such potential donors.
- Other lineages of Bacteria and Archaea including the ancestors of *T. tepidarius*, once contained the genes but all subsequently lost them except the *Acidithiobacillus* genus. We think that this is the least likely explanation because it requires many independent gene loss events to have occurred. Also, if the proposed association of families I–V with functions involved in acid related response is correct, it would suggest that many ancestral lineages of the *Acidithiobacillus* genus were acidophiles for which there is no evidence.

Although a lack of definitive evidence leaves all three hypothesis unimpaired, we speculate that the emergence of families I–V could have helped promote by whatever means (direct activity of the encoded proteins, or via sensing or regulatory mechanisms) the ability of the last common ancestor of the *Acidithiobacillus* genus and *T. tepidarius* to transition from a neutral pH environment to one that was increasingly acidic and finally to one that was extremely acidic. In this scenario, the transition process could have provided opportunities for the *Acidithiobacillus* genus to diverge from the *T. tepidarius* lineage. This hypothesis requires additional evidence, especially experimental evidence, to clearly pinpoint the specific functions and physiological roles of the five families.

### Use of families I–V as genetic probes for *Acidithiobacillus* genus and species identification

**Figure 5:**
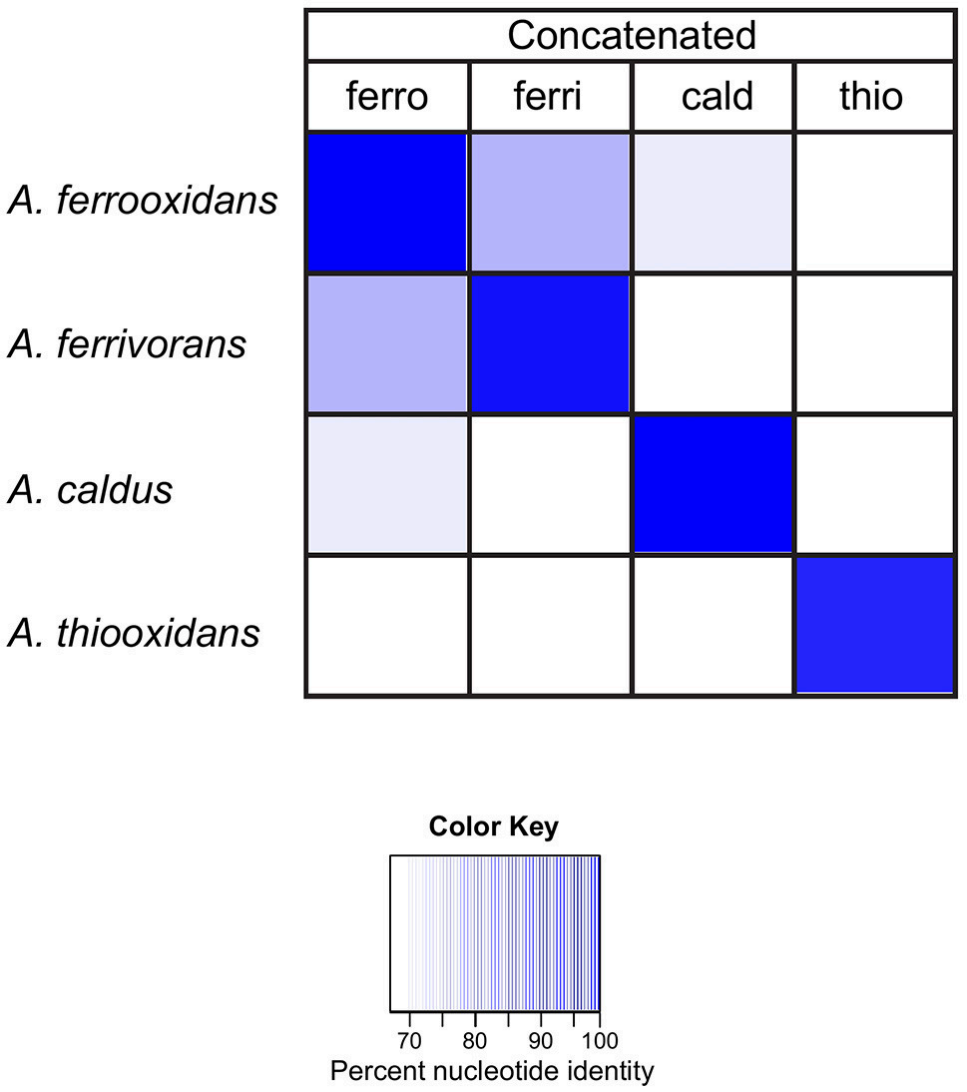
**Heat map showing the percent nucleotide similarity (from 100% to <70%, see color key) between families I–V genes, concatenated for each ***Acidithiobacillus*** species (top row) vs. families I–V in each genome (left)**. ferro: *A*. *ferrooxidans*; ferri: *A*. *ferrivorans*; thio: *A*. *thiooxidans* and cald: *A*. *caldus*.

In order to evaluate the sensitivity and specificity of the families to discriminate between *Acidithiobacillus* species, the DNA sequences of families I–V were concatenated for each *Acidithiobacillus* species and compared by BLASTN against each *Acidithiobacillus* species. The results are reported as % nucleotide identity between the concatenated probe and each *Acidithiobacillus* species (Figure 5). The dark blue diagonal indicates high nucleotide identity, as expected, between the concatenated probe and its respective sequences in the corresponding genome. Importantly, the concatenated probes from one species have lower levels of sequence identity when compared to other species. For example, the concatenated probe from *A. caldus* has only 69% identity (white cell) when compared to sequences present in the genome of *A. ferrivorans*.

These data indicate that the concatenated families are capable of discriminating between the different *Acidithiobacilli* species used to build the concatenated probes, but are they capable of phylotyping new genomes that did not contribute to building the probes?

During the course of this investigation four new genomes of *A. ferrooxidans* (strains BY0502, DLC-5, YQH-1, and Hel18), one *A. caldus* genome (strain MTH-04) and six genomes of *A. thiooxidans* were released (Table 1), providing an opportunity to test the discriminatory powers of the family probes on new genomes.

**Figure 6:**
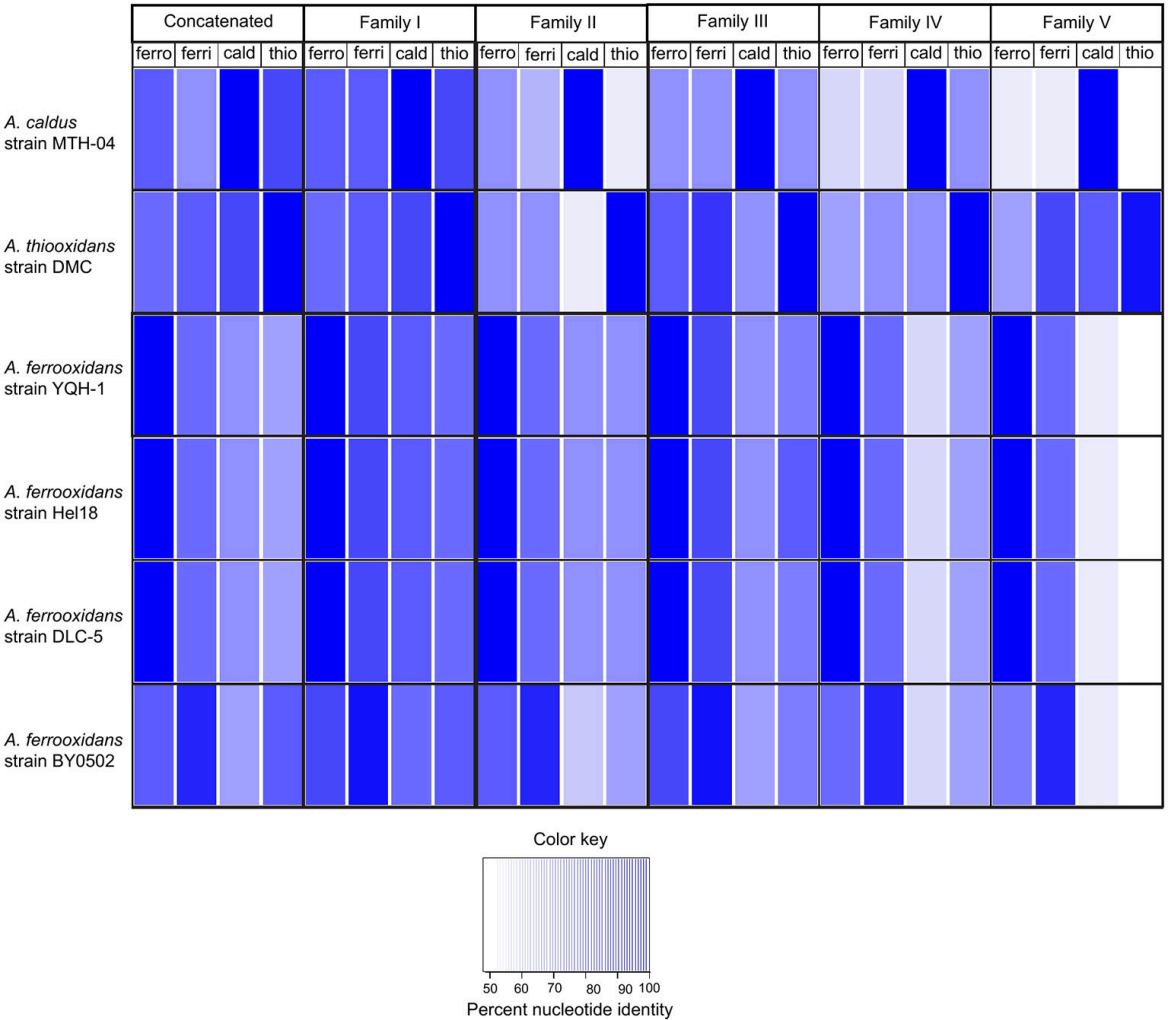
**Heat map illustrating the percent nucleotide similarity (from 100% to <50%, see color key) between families I–V genes and the best BLAST hit of four newly identified ***A. ferrooxidans*** genomes (strain BY0502, strain DLC-5, strain YQH-1, and strain Hel18) and one ***A. caldus*** genome (strain MTH-04)**.

First, the concatenated family probes, described in the previous experiment, were used in BLASTN comparisons with the new genomes. The results are reported as % nucleotide identity between the concatenated probe and each *Acidithiobacillus* species (leftmost four columns, Figure 6). The concatenated probes clearly have the ability to discriminate between *A. caldus* MTH-04, *A. thiooxidans* DMC, *A. ferrooxidans* BY0502, *A. ferrooxidans* YQH-1, and *A. ferrooxidans* Hel18, indicated by the dark blue color (close to 100% sequence identity). However, there is one anomalous identification. *A. ferrooxidans* BY0502 exhibits the best match with the *A. ferrivorans* concatenated probe (bottom row), suggesting that this species might not be *A. ferrooxidans*.

In order to determine if this anomaly could be attributed to one (or more) of the families in particular, the experiment was repeated with each individual family (Figure 6). Each family correctly identified the new genomes of *A. ferrooxidans, A. thiooxidans* and *A. caldus* with the exception of *A. ferrooxidans* BY0502. The highest percentage matches of all five families to *A. ferrooxidans* BY0502 were to the probes built from *A. ferrivorans*, confirming the results using the concatenated family probe.

**Figure 7:**
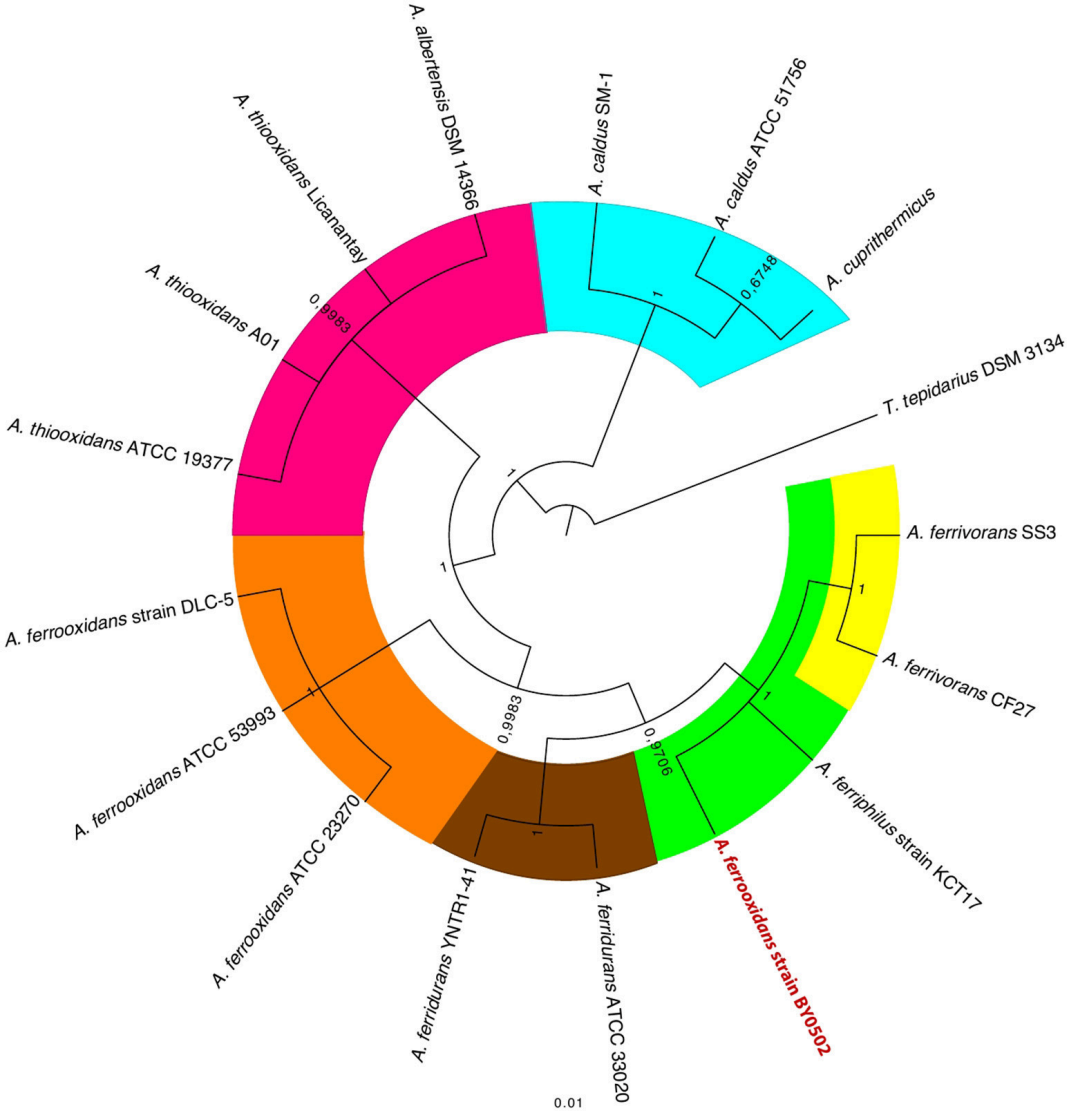
**16S rRNA gene tree of selected ***Acidithiobacilli*** showing the anomalous location of ***A. ferrooxidans*** BY0502 within the ***A. ferriphilus*** clade**. The tree was constructed using bayesian inference with MrBayes (Huelsenbeck and Ronquist, 2001). The posterior probability node support is given for all nodes.

Because of the vexing problem of the anomalous *A. ferrooxidans* BY0502 in which the family I–V probes place it closer to *A. ferrivorans* than *A. ferrooxidans*, it was decided to use other approaches to investigate its phylogeny using ANI (Goris et al., 2007) and TETRA (Richter and Rosselló-Móra, 2009). Both approaches indicate that *A. ferrooxidans* BY0502 is not related to *A. ferrooxidans* because of the low values of ANI and TETRA, 83.4 and 0.988, respectively, between the two genomes. Nor can it be classified in the *A. ferrivorans* clade, with low values of 91.7/0.996 (ANI/TETRA values), although it is more closely related to *A. ferrivorans* than *A. ferrooxidans*. In order to investigate further the phylogeny of *A. ferrooxidans* BY0502, 16S rRNA sequence analysis was carried out that placed it in a clade with *A. ferriphilus*, subtended by the clade *A. ferrivorans* with a bayesian posterior probability node support of 1 that strongly endorses the proposed phylogeny (Figure 7). Therefore, we suggest that *A. ferrooxidans* BY0502 is more likely to be an *A. ferriphilus-like* microorganism; an hypothesis that requires confirmation using other phylogenetic approaches. This example demonstrates the power of the family probes to aid in the identification of the *Acidithiobacillus* genus with discriminatory powers to suggest species at least for those under interrogation in the present study.

### Use of families I–V as genetic probes for interrogation of metagenomes and metatranscriptomes

Gaining insight into the structure, organization, and function of microbial communities (microbiomes) has been proposed as one of the major research challenges of the current decade (2020 visions, 2010) and metagenomic and metatranscriptomic approaches present major opportunities for advancing our knowledge in this area. One of the most promising areas of metagenomics research is the use of shotgun methods to sequence random fragments of DNA (or RNA) in an environmental sample. This information can then be analyzed for microbial diversity, prediction of gene functions and biochemical pathway model building. Many bioinformatic approaches have been developed to handle the typically enormous amounts of data generated by metagenomics investigations (e.g., reviewed in Hiraoka et al., 2016).

One of the most straightforward and computationally less demanding approaches to estimate microbial diversity in a microbiome is the use of marker genes (molecular probes; Wu and Eisen, 2008; Liu et al., 2011; Wu and Scott, 2012; Kim et al., 2013; Darling et al., 2014). For example, rRNA sequences from known organisms can be used to computationally search the shotgun sequences for similar sequences or can be coupled with rRNA-PCR to pull out and extend specific sequences. These methods provide an overview of the phylogenetic distribution (phylotyping) of the cell-based life present in a sample but they have their limitations (reviewed in Fabrice and Didier, 2009).

Taxonomically restricted protein encoding genes have been used for phylotyping, including the recombinase A gene family and the RNA polymerase beta subunit (Wu et al., 2011), genes specifically targeting the *Acidithiobacilli* (Nieto et al., 2009; Nuñez et al., 2014, 2016) and many other examples (Liu et al., 2011; Segata et al., 2011; Wu et al., 2013; Darling et al., 2014). However, such marker genes are subject to HGT and evolutionary rate differences that can exacerbate the interpretation of phylogenies. Since the five families are taxonomically restricted to the *Acidithiobacilli* and do not appear to be prone to HGT, we decided to examine their ability to identify the *Acidithiobacillus* genus and to discriminate between different species of the *Acidithiobacilli* (Figures 6, 7) in environmental metagenomic and metatranscriptomic samples. For the first objective, the amino acid sequence of all five families from all participating *Acidithiobacillus* species (*A. ferrooxidans, A. ferrivorans, A. thiooxidans*, and *A. caldus*) was concatenated (five families × nine species). This was considered as a general probe for the *Acidithiobacillus* genus (genus-level probe). A second series of probes was constructed where the protein sequences of the five families was concatenated according to species, generating five different probes each one specific for an *Acidithiobacillus* species (e.g., *A. ferrooxidans* probe = the concatenation of families I–V of *A. ferrooxidans*). These probes were then used in a BLASTX searches to interrogate several environmental metagenomes and metatranscriptomes listed in Table 4.

**Table 4:** **Detection of ***Acidithiobacilli*** in (A) various metagenomes and (B) metatranscriptomes using families I–V as molecular probes**.

**(A) Study name**	**Sample type**	**Source**	**pH**	**Database source**	**ID**	***Acidithiobacilli*** **reported**	***Acidithiobacilli*** **detected using Families I–V (this study) in reference**
Kristineberg Mine	P	Malå, Sweden	2.5–2.7	NCBI nr	AOMQ00000000	AFV, AFE, ATHIO, ACAL (Liljeqvist et al., 2015)	AFV, AFE, ATHIO, ACAL
Kristineberg Mine	B	Malå, Sweden	2.5–2.7	NCBI nr	AOMP00000000	AFV, AFE, ATHIO, ACAL (Liljeqvist et al., 2015)	AFV, AFE, ATHIO, ACAL
Pink biofilm Richmond Mine	AMD	California, USA	0.83	NCBI nr	AADL00000000	None (Tyson et al., 2004)	Not detected
Carnoulès Mine (bin 5)	AMD	Gard, France	3.5–3.8	NCBI nr	PRJNA62261	AFE (Bertin et al., 2011)	AFE, ATHIO, ACAL
Snottites in Frasassi Cave	AMD	Ancona, Italy	0–1	NCBI nr	SRP006444	ATHIO, AT (Jones et al., 2012)	ATHIO
Acquasanta Terme AS5	SB	Grotta Nuova di Rio Garrafo, Italy	0–1.5	IMG/M	3300000825	ATHIO (Jones et al., 2016)	ATHIO
Black Soud Mine	AMD	Minnesota, USA	6.7	NCBI nr	ABLV00000000	None (Edwards et al., 2006)	Not detected
Black smokers (Tui Malila)	HVP	Lau Basin, Pacific Ocean	3.8–5.7	IMG/M	3300001676	None (Sheik et al., 2015)	Not detected
Hydrothermal vent (Guaymas Basin)	HVP	Guaymas Basin, Pacific Ocean	6.5–8	IMG/M	3300003086	None (Li et al., 2016)	Not detected
Marine Microbial communities (Loihi)	HVP	Loihi Seamount, Hawaii	8	IMG/M	3300000327	None (Singer et al., 2013)	Not detected
Deep Oceanic Microbial Communities (Juan de Fuca)	HVP	Juan de Fuca, Pacific Ocean	4.2	IMG/M	3300002481	None (Jungbluth et al., 2013)	Not detected
Marine Microbial communities (Lost City)	HVP	Lost City, Atlantic Ocean	9–11	IMG/M	3300003136	None (Anantharaman et al., 2014)	Not detected
**(B) Study**	**Sample type**	**Origin**	**pH**	**Database source**	**ID**	***Acidithiobacillus*** **reported**	***Acidithiobacillus*** **detected with family probes in reference**
Dabaoshan Mine	AMD	Guangdong, China	1.9–2.3	MG-RAST	4481316.3	AFE, AFV (Chen et al., 2015)	AFE, AFV, ATHIO
Yunfu Mine	AMD	Guangdong, China	2.5	MG-RAST	4481318.3	AFE, AFV (Chen et al., 2015)	AFE, AFV, ATHIO

*AMD, Acid Mine Drainage; ACAL, A. caldus; AFV, A. ferrivorans; AFE, A. ferrooxidans; ATHIO, A. thiooxidans; AT, Acidithiobacillus genus; P, Planktonic; B, Biofilm; SB, Subaerial biofilm; HVP, Hydrothermal vent plume; NCBI nr, National Center for Biotechnology Information, non-redundant database; IMG/M, Integrated Microbial Genomes/ Metagenomes; MG-RAST, Metagenomes- Rapid Annotation using Subsystem Technology*.

**Figure 8:**
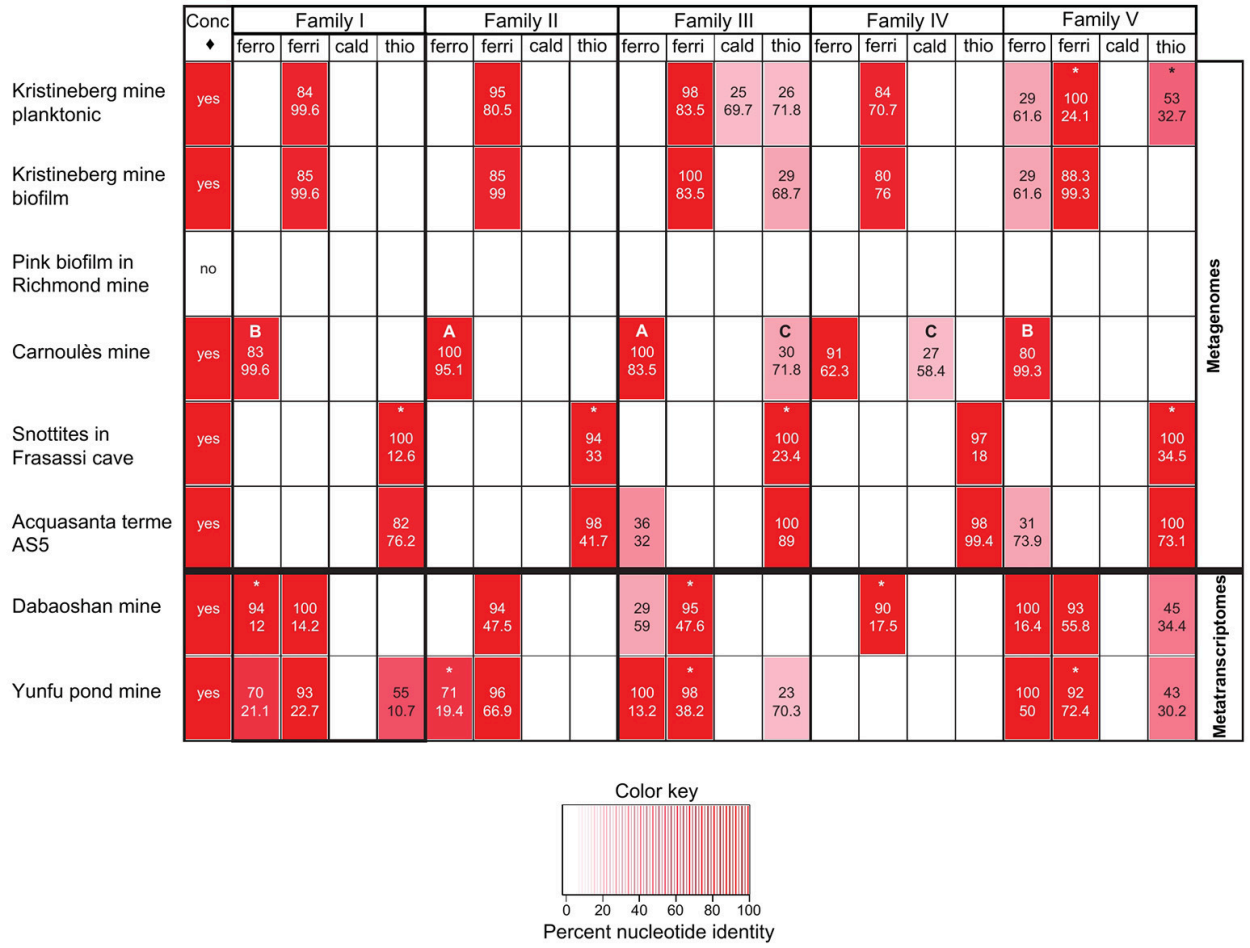
**Heat map indicating the percent nucleotide identity (top number in respective cells) and sequence coverage (lower number in respective cells) between families I–V and environmental metagenomes and metatranscriptomes as assayed by BLASTX**. The figure also shows (leftmost column, ♢ = concatenated probe) the presence or absence of the *Acidithiobacillus* genus in the metagenomes and metatranscriptomes determined by BLASTX, using as a probe the concatenated sequences of all five families of all *Acidithiobacilli* used in the study (5 families × 9 *Acidithiobacilli* species = 45 concatenated sequences), where positive matching is indicated with a “yes.” The letters A to C refer to specific cases described in the text. The ^*^ refers to sequences that are truncated in the respective metagenome/transcriptome databases.

The metagenomes were chosen to include low pH environments such as mining operations and AMD, where *Acidithiobacilli* have previously been reported, and also environments of intermediate acidity (e.g., Black Smokers, Tui Malila), neutral pH (e.g., Hydrothermal vent, Guaymas Basin), and high pH (e.g., Marine Microbial Communities, Lost City) where *Acidithiobacilli* have not been detected. Two low pH metatranscriptomes were also included in the analysis. The results of the BLASTX interrogations are shown in Figure 8 and the results are summarized in Table 4.

Inspection of the left hand column of Figure 8 indicates that the genus-level probe detects sequence similarity in all the samples except for the Pink Biofilm from the Richmond mine. This is in agreement with the report that no *Acidithiobacilli* were detected in the Pink Biofilm but were detected in all the other samples (references provided in Table 4). The absence of *Acidithiobacilli* in the Pink Biofilm sample could be due to its extremely low pH (pH 0.83) which is thought to be too acidic to support their growth (Tyson et al., 2004). In addition no *Acidithiobacilli* were detected in samples from the Black Soud Mine, Black Smokers (Tui Malila), Hydrothermal Vent (Guaymas Basin), Marine Microbial Communities (Loihi), Deep Ocean Microbial Communities (Juan de Fuca), Marine Microbial Communities (Lost City), which is also in agreement with the published literature (references found in Table 4). The conclusion is that the *Acidithiobacilli* genus-level probe appears to have good specificity and sensitivity in detecting *Acidithiobacilli* in environmental metagenomes but more samples are required to develop statistical support for this assertion.

Table 4 also indicates that the families can be used to interrogate metatranscriptomes and provides additional evidence that the genes of family I–V are transcribed. This evidence was used to construct the right hand column presented earlier in Table 3.

However, caution is required in the interpretation of the use of the species-specific probes. In case A (see Figure 8), both the sequence identity (100%) and sequence coverage (83.5–95.1%) of the *A. ferrooxidans* probes of families II and III strongly support the contention that sequences corresponding to them are present in the Carnoulès metagenome. However, in case B, although there is good coverage of the *A. ferrooxidans* family I and V probes (99.3–99.6%), the sequence identity is lower (80–83%). This suggests that these families probably belong to *A. ferrooxidans* in the metagenome but that they have diverged somewhat from the probe sequences. Recovery of such sequences would expand the number and diversity of such sequences that could be helpful for elucidating their function and shedding light on their evolution. In case C, both the coverage and identity are lower and the hits are to probes developed for *A. thiooxidans* and *A. caldus* family III and family IV. This suggests that the Carnoulès metagenome contains *A. thiooxidans*-like and *A. caldus*-like organisms that exhibit low sequence similarity to families III and IV, but not to the other families. As in case B, these sequences could be helpful for later studies to help unravel sequence function and evolution. A final case marked by asterisks in Figure 8 illustrates the common finding of sequence similarity to metagenomic reads that are truncated. Truncated sequences that have high similarity to the probes could potentially be extended by PCR using primers designed from the probes and subsequently analyzed.

With these caveats in mind, families I–V satisfy a number of criteria for use as identification markers for *Acidithiobacilli* in genomic, metagenomic/metatranscriptomic investigations. They are universally present in the genus, not present in other genera and are not subject to HGT. Preliminary evidence also points to association of at least three of the families (Families I, III, and IV) in envelope remodeling and lipid metabolism possibly associated with acid stress response and so could serve as PhyEco (for phylogenetic and phylogenetic ecology; Wu et al., 2013) markers for certain acidic environments including AMD and biomining operations.

## Conclusions

This study:

- Used comparative genomics approaches to discover five protein families that are taxonomically restricted to the genus *Acidithiobacillus* (*Acidithiobacilli*), a group of extreme acidophiles.
- Highlighted and examined the potential functions of the five families. Although functional assignments could not be made with confidence for any of the families, it was hypothesized that they are involved in cell envelope restructuring that in four families may be associated with responses to changing pH conditions, at least in *A. caldus*.
- Reflected on the possible evolution of the five families. It was suggested that the five families emerged after the split of the *Acidithiobacilli* lineage from the neutrophile *T. tepidarius*, allowing the *Acidithiobacilli* lineage to colonize acidic econiches.
- Considered how the five families can be used as molecular probes to interrogate genomic and metagenomic/metatranscriptomic data.
- Served as a springboard for testing hypotheses and for guiding future research, for example to: (i) investigate experimentally the hypothesis that some of the orphan family genes could be involved in acid stress response(s) and/or membrane remodeling, (ii) explore further the concept that the orphan family genes have played a role in the evolution of the *Acidithiobacilli* from neutral ancestors to modern day extreme acidophiles, and (iii) use additional tools to investigate the phylogeny of *A. ferrooxidans* BY0502 that our study suggests is more likely to be a *Ferriphilus*-like microorganism.

## Future perspectives

As more data become available from genomic and metagenome sequencing projects, it will be possible to determine if families I–V maintain their ability to be specific probes for the genus *Acidithiobacillus*. The availability of additional examples of families I–V could advance our understanding of their function, origin and evolutionary trajectory.

## Author contributions

DH and JV conceived the project. DH and CG designed the experiments. ML and CG carried out the experiments. All authors analyzed the data. DH drafted the manuscript. All authors contributed to subsequent drafts of the manuscript. All authors read and approved the final manuscript.

### Conflict of interest statement

The authors declare that the research was conducted in the absence of any commercial or financial relationships that could be construed as a potential conflict of interest.

## Footnotes

^1^http://linux1.softberry.com/berry.phtml?topic=pcompb&group=programs&subgroup=proloc
