# Supplementary material for: Bioinformatic Analyses of Unique (Orphan) Core Genes of the Genus Acidithiobacillus: Functional Inferences and Use As Molecular Probes for Genomic and Metagenomic/Transcriptomic Interrogation
Source: Front Microbiol. 2016 Dec 27;7:2035. doi: 10.3389/fmicb.2016.02035 (PMC5186765; doi:10.3389/fmicb.2016.02035)
Supplement: Supplementary file 1 [file DataSheet1.pdf]

SUPPLEMENTAL FILE 1

Protein and DNA Alignments of Families I-V

Bioinformatic Analyses of Unique (Orphan) Core Genes of the Genus *Acidithiobacillus*: Functional Inferences and Use As Molecular Probes for Genomic and Metagenomic/Transcriptomic Interrogation

Carolina González, Marcelo Lazcano, Jorge Valdés and David S. Holmes

frontiers in Microbiology

Family I

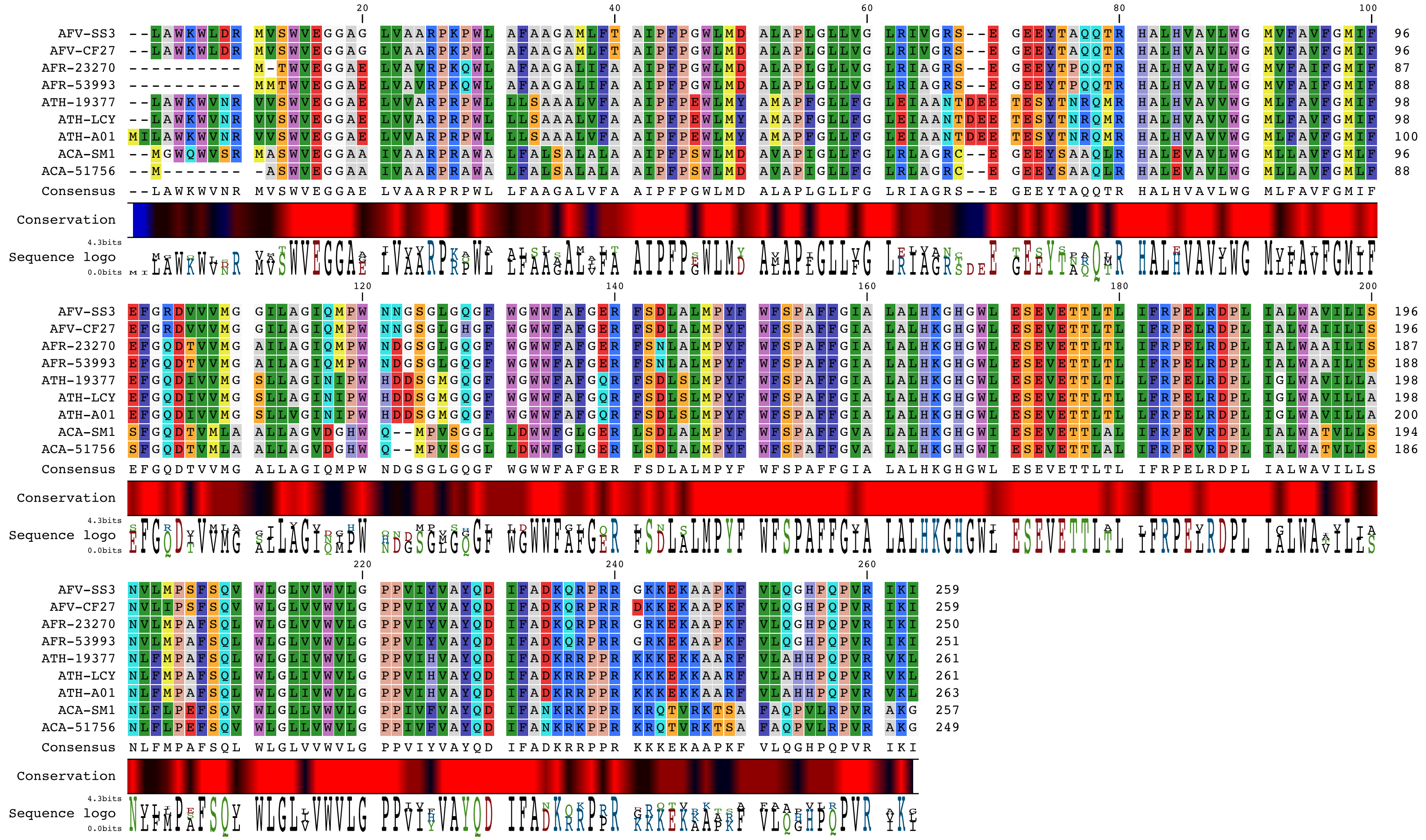
AFV-SS3 --LAWKWLDR MVSWVEGGAG LVAARPKPWL AFAAGAMLFT AIPFPGWLMD ALAPLGLLVG LRIVGRS--E GEEYTAQQTR HALHVAVLWG MVFAVFGMIF 96
AFV-CF27 --LAWKWLDR MVSWVEGGAG LVAARPKPWL AFAAGAMLFT AIPFPGWLMD ALAPLGLLVG LRIVGRS--E GEEYTAQQTR HALHVAVLWG MVFAVFGMIF 96
AFR-23270 ---------- M-TWVEGGAE LVAVRPKQWL AFAAGALIFA AIPFPGWLMD ALAPLGLLVG LRIAGRS--E GEEYTPQQTR HALHVAVLWG MVFAIFGMIF 87
AFR-53993 ---------- MMTWVEGGAE LVAVRPKQWL AFAAGALIFA AIPFPGWLMD ALAPLGLLVG LRIAGRS--E GEEYTPQQTR HALHVAVLWG MVFAIFGMIF 88
ATH-19377 --LAWKWVNR VVSWVEGGAE LVVARPRPWL LLSAAALVFA AIPFPEWLMY AMAPFGLLFG LEIAANTDEE TESYTNRQMR HALHVAVVWG MLFAVFGMIF 98
ATH-LCY --LAWKWVNR VVSWVEGGAE LVVARPRPWL LLSAAALVFA AIPFPEWLMY AMAPFGLLFG LEIAANTDEE TESYTNRQMR HALHVAVVWG MLFAVFGMIF 98
ATH-A01 MILAWKWVNR VVSWVEGGAE LVVARPRPWL LLSAAALVFA AIPFPEWLMY AMAPFGLLFG LEIAANTDEE TESYTNRQMR HALHVAVVWG MLFAVFGMIF 100
ACA-SM1 --MGWQWVSR MASWVEGGAA IVAARPRAWA LFALSALALA AIPFPSWLMD AVAPIGLLFG LRLAGRC--E GEEYSAAQLR HALEVAVLWG MLLAVFGMLF 96
ACA-51756 --M------- -ASWVEGGAA IVAARPRAWA LFALSALALA AIPFPSWLMD AVAPIGLLFG LRLAGRC--E GEEYSAAQLR HALEVAVLWG MLLAVFGMLF 88
Consensus --LAWKWVNR MVSWVEGGAE LVAARPRPWL LFAAGALVFA AIPFPGWLMD ALAPLGLLFG LRIAGRS--E GEEYTAQQTR HALHVAVLWG MLFAVFGMIF
Conservation
Sequence logo
4.3bits
0.0bits
AFV-SS3 EFGRDVVVMG GILAGIQMPW NNGSGLGQGF WGWWFAFGER FSDLALMPYF WFSPAFFGIA LALHKGHGWL ESEVETTLTL IFRPELRDPL IALWAVILIS 196
AFV-CF27 EFGRDVVVMG GILAGIQMPW NNGSGLGHGF WGWWFAFGER FSDLALMPYF WFSPAFFGIA LALHKGHGWL ESEVETTLTL IFRPELRDPL IALWAIILIS 196
AFR-23270 EFGQDTVVMG AILAGIQMPW NDGSGLGQGF WGWWFAFGER FSNLALMPYF WFSPAFFGIA LALHKGHGWL ESEVETTLTL IFRPELRDPL IALWAAILIS 187
AFR-53993 EFGQDTVVMG AILAGIQMPW NDGSGLGQGF WGWWFAFGER FSNLALMPYF WFSPAFFGIA LALHKGHGWL ESEVETTLTL IFRPELRDPL IALWAAILIS 188
ATH-19377 EFGQDIVVMG SLLAGINIPW HDDSGMGQGF WGWWFAFGQR FSDLSLMPYF WFSPAFFGIA LALHKGHGWL ESEVETTLTL LFRPELRDPL IGLWAVILLA 198
ATH-LCY EFGQDIVVMG SLLAGINIPW HDDSGMGQGF WGWWFAFGQR FSDLSLMPYF WFSPAFFGIA LALHKGHGWL ESEVETTLTL LFRPELRDPL IGLWAVILLA 198
ATH-A01 EFGQDIVVMG SLLVGINIPW HDDSGMGQGF WGWWFAFGQR FSDLSLMPYF WFSPAFFGIA LALHKGHGWL ESEVETTLTL LFRPELRDPL IGLWAVILLA 200
ACA-SM1 SFGQDTVMLA ALLAGVDGHW Q--MPVSGGL LDWWFGLGER LSDLALMPYF WFSPAFFGVA LALHKGHGWI ESEVETTLAL IFRPEVRDPL IALWATVLLS 194
ACA-51756 SFGQDTVMLA ALLAGVDGHW Q--MPVSGGL LDWWFGLGER LSDLALMPYF WFSPAFFGVA LALHKGHGWI ESEVETTLAL IFRPEVRDPL IALWATVLLS 186
Consensus EFGQDTVVMG ALLAGIQMPW NDGSGLGQGF WGWWFAFGER FSDLALMPYF WFSPAFFGIA LALHKGHGWL ESEVETTLTL IFRPELRDPL IALWAVILLS
AFV-SS3 NVLMPSFSQV WLGLVVWVLG PPVIYVAYQD IFADKQRPRR GKKEKAAPKF VLQGHPQPVR IKI 259
AFV-CF27 NVLIPSFSQV WLGLVVWVLG PPVIYVAYQD IFADKQRPRR DKKEKAAPKF VLQGHPQPVR IKI 259
AFR-23270 NVLMPAFSQL WLGLVVWVLG PPVIYVAYQD IFADKQRPRR GRKEKAAPKF VLQGHPQPVR IKI 250
AFR-53993 NVLMPAFSQL WLGLVVWVLG PPVIYVAYQD IFADKQRPRR GRKEKAAPKF VLQGHPQPVR IKI 251
ATH-19377 NLFMPAFSQL WLGLIVWVLG PPVIHVAYQD IFADKRRPPR KKKEKKAARF VLAHHPQPVR VKL 261
ATH-LCY NLFMPAFSQL WLGLIVWVLG PPVIHVAYQD IFADKRRPPR KKKEKKAARF VLAHHPQPVR VKL 261
ATH-A01 NLFMPAFSQL WLGLIVWVLG PPVIHVAYQD IFADKRRPPR KKKEKKAARF VLAHHPQPVR VKL 263
ACA-SM1 NLFLPEFSQV WLGLLVWVLG PPIVFVAYQD IFANKRKPPR KRQTVRKTSA FAQPVLRPVR AKG 257
ACA-51756 NLFLPEFSQV WLGLLVWVLG PPIVFVAYQD IFANKRKPPR KRQTVRKTSA FAQPVLRPVR AKG 249
Consensus NLFMPAFSQL WLGLVVWVLG PPVIYVAYQD IFADKRRPPR KKKEKAAPKF VLQGHPQPVR IKI

Family II

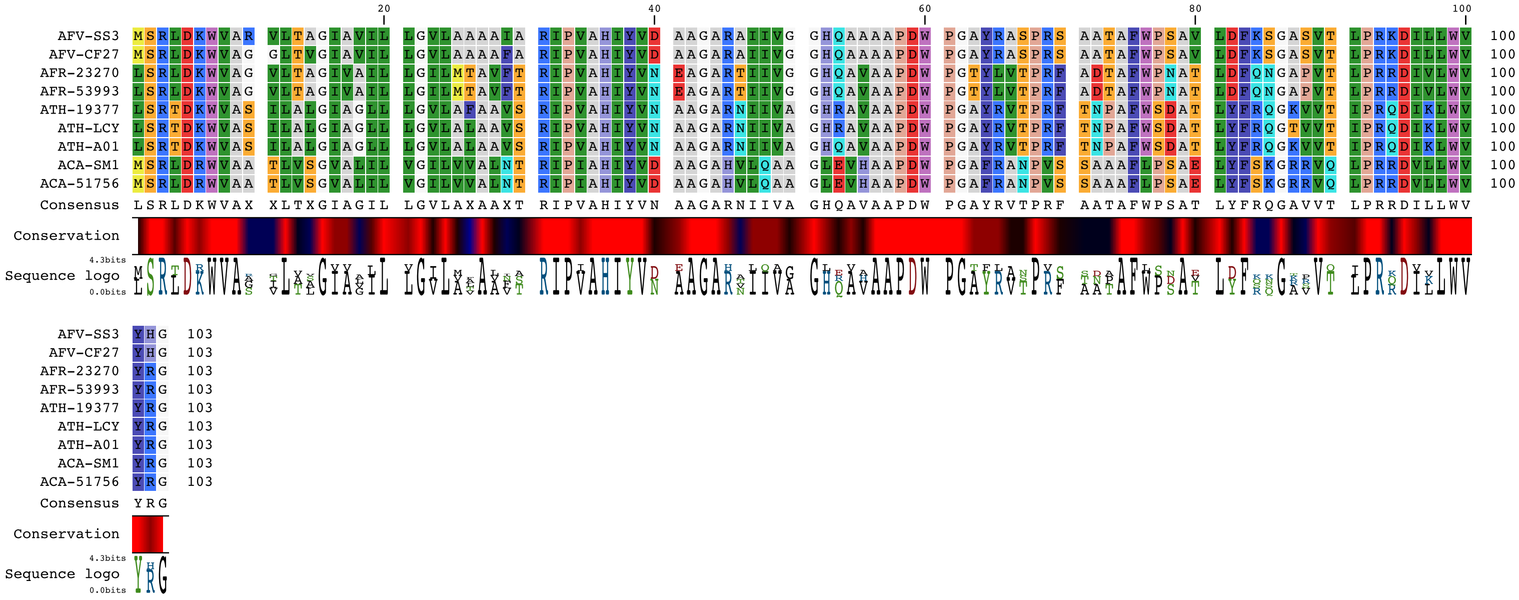
20 40 60 80 100
AFV-SS3 MSRLDKWVAR VLTAGIAVIL LGVLAAAAIA RIPVAHIYVD AAGARAIIVG GHQAAAAPDW PGAYRASPRS AATAFWPSAV LDFKSGASVT LPRKDILLWV 100
AFV-CF27 MSRLDKWVAG GLTVGIAVIL LGVLAAAAFA RIPVAHIYVD AAGARAIIVG GHQAAAAPDW PGAYRASPRS AATAFWPSAV LDFKSGASVT LPRKDILLWV 100
AFR-23270 LSRLDKWVAG VLTAGIVAIL LGILMTAVFT RIPVAHIYVN EAGARTIIVG GHQAVAAPDW PGTYLVTPRF ADTAFWPNAT LDFQNGAPVT LPRRDIVLWV 100
AFR-53993 LSRLDKWVAG VLTAGIVAIL LGILMTAVFT RIPVAHIYVN EAGARTIIVG GHQAVAAPDW PGTYLVTPRF ADTAFWPNAT LDFQNGAPVT LPRRDIVLWV 100
ATH-19377 LSRTDKWVAS ILALGIAGLL LGVLAFAAVS RIPVAHIYVN AAGARNIIVA GHRAVAAPDW PGAYRVTPRF TNPAFWSDAT LYFRQGKVVT IPRQDIKLWV 100
ATH-LCY LSRTDKWVAS ILALGIAGLL LGVLALAAVS RIPVAHIYVN AAGARNIIVA GHRAVAAPDW PGAYRVTPRF TNPAFWSDAT LYFRQGTVVT IPRQDIKLWV 100
ATH-A01 LSRTDKWVAS ILALGIAGLL LGVLALAAVS RIPVAHIYVN AAGARNIIVA GHQAVAAPDW PGAYRVTPRF TNPAFWSDAT LYFRQGKVVT IPRQDIKLWV 100
ACA-SM1 MSRLDRWVAA TLVSGVALIL VGILVVALNT RIPIAHIYVD AAGAHVLQAA GLEVHAAPDW PGAFRANPVS SAAAFLPSAE LYFSKGRRVQ LPRRDVLLWV 100
ACA-51756 MSRLDRWVAA TLVSGVALIL VGILVVALNT RIPIAHIYVD AAGAHVLQAA GLEVHAAPDW PGAFRANPVS SAAAFLPSAE LYFSKGRRVQ LPRRDVLLWV 100
Consensus LSRLDKWVAX XLTXGIAGIL LGVLAXAAXT RIPVAHIYVN AAGARNIIVA GHQAVAAPDW PGAYRVTPRF AATAFWPSAT LYFRQGAVVT LPRRDILLWV
Conservation
4.3bits
Sequence logo
0.0bits
AFV-SS3 YHG 103
AFV-CF27 YHG 103
AFR-23270 YRG 103
AFR-53993 YRG 103
ATH-19377 YRG 103
ATH-LCY YRG 103
ATH-A01 YRG 103
ACA-SM1 YRG 103
ACA-51756 YRG 103
Consensus YRG
Conservation
4.3bits
Sequence logo
0.0bits

Family III

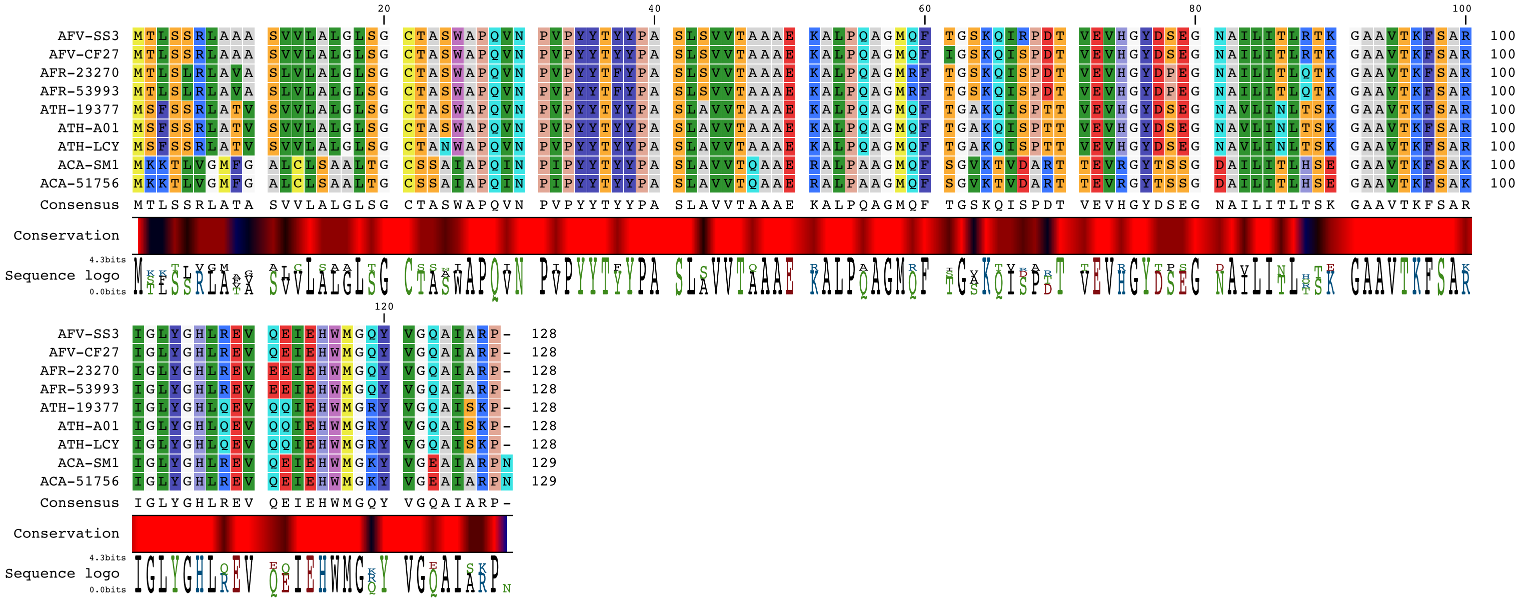
20
40
60
80
100
AFV-SS3 MTLSSRLAAA SVVLALGLSG CTASWAPQVN PVPYYTYYPA SLSVVTAAAE KALPQAGMQF TGSKQIRPDT VEVHGYDSEG NAILITLRTK GAAVTKFSAR 100
AFV-CF27 MTLSSRLAAA SVVLALGLSG CTASWAPQVN PVPYYTYYPA SLSVVTAAAE KALPQAGMQF IGSKQISPDT VEVHGYDSEG NAILITLRTK GAAVTKFSAR 100
AFR-23270 MTLSLRLAVA SLVLALGLSG CTASWAPQVN PVPYYTFYPA SLSVVTAAAE KALPQAGMRF TGSKQISPDT VEVHGYDPEG NAILITLQTK GAAVTKFSAR 100
AFR-53993 MTLSLRLAVA SLVLALGLSG CTASWAPQVN PVPYYTFYPA SLSVVTAAAE KALPQAGMRF TGSKQISPDT VEVHGYDPEG NAILITLQTK GAAVTKFSAR 100
ATH-19377 MSFSSRLATV SVVLALGLSG CTASWAPQVN PVPYYTYYPA SLAVVTAAAE KALPQAGMQF TGAKQISPTT VEVHGYDSEG NAVLINLTSK GAAVTKFSAR 100
ATH-A01 MSFSSRLATV SVVLALGLSG CTASWAPQVN PVPYYTYYPA SLAVVTAAAE KALPQAGMQF TGAKQISPTT VEVHGYDSEG NAVLINLTSK GAAVTKFSAR 100
ATH-LCY MSFSSRLATV SVVLALGLSG CTANWAPQVN PVPYYTYYPA SLAVVTAAAE KALPQAGMQF TGAKQISPTT VEVHGYDSEG NAVLINLTSK GAAVTKFSAR 100
ACA-SM1 MKKTLVGMFG ALCLSAALTG CSSAIAPQIN PIPYYTYYPA SLAVVTQAAE RALPAAGMQF SGVKTVDART TEVRGYTSSG DAILITLHSE GAAVTKFSAK 100
ACA-51756 MKKTLVGMFG ALCLSAALTG CSSAIAPQIN PIPYYTYYPA SLAVVTQAAE RALPAAGMQF SGVKTVDART TEVRGYTSSG DAILITLHSE GAAVTKFSAK 100
Consensus MTLSSRLATA SVVLALGLSG CTASWAPQVN PVPYYTYYPA SLAVVTAAAE KALPQAGMQF TGSKQISPDT VEVHGYDSEG NAILITLTSK GAAVTKFSAR
Conservation
4.3bits
Sequence logo
0.0bits
120
AFV-SS3 IGLYGHLREV QEIEHWMGQY VGQAIARP- 128
AFV-CF27 IGLYGHLREV QEIEHWMGQY VGQAIARP- 128
AFR-23270 IGLYGHLREV EEIEHWMGQY VGQAIARP- 128
AFR-53993 IGLYGHLREV EEIEHWMGQY VGQAIARP- 128
ATH-19377 IGLYGHLQEV QQIEHWMGRY VGQAISKP- 128
ATH-A01 IGLYGHLQEV QQIEHWMGRY VGQAISKP- 128
ATH-LCY IGLYGHLQEV QQIEHWMGRY VGQAISKP- 128
ACA-SM1 IGLYGHLREV QEIEHWMGKY VGEAIARPN 129
ACA-51756 IGLYGHLREV QEIEHWMGKY VGEAIARPN 129
Consensus IGLYGHLREV QEIEHWMGQY VGQAIARP-
Conservation
4.3bits
Sequence logo
0.0bits

Family IV

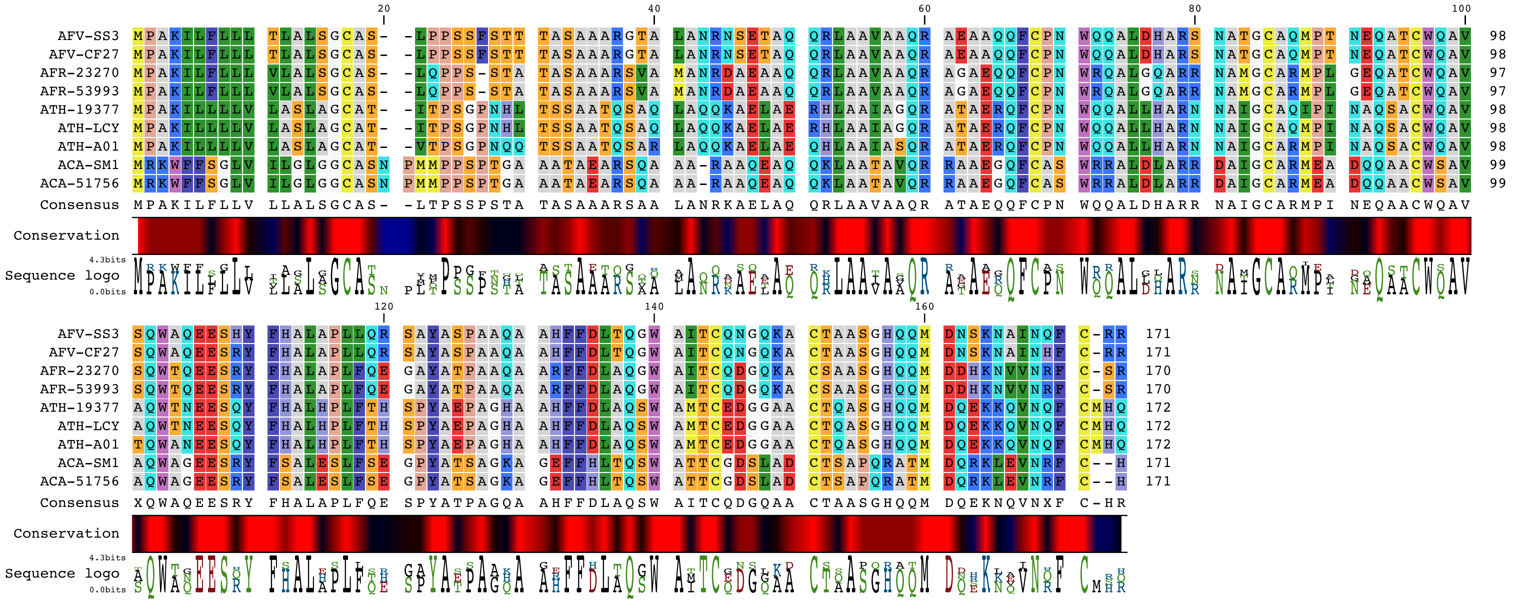
20 40 60 80 100
AFV-SS3 MPAKILFLLL TLALSGCAS- -LPPSSFSTT TASAAARGTA LANRNSETAQ QRLAAVAAQR AEAAQQFCPN WQQALDHARS NATGCAQMPT NEQATCWQAV 98
AFV-CF27 MPAKILFLLL TLALSGCAS- -LPPSSFSTT TASAAARGTA LANRNSETAQ QRLAAVAAQR AEAAQQFCPN WQQALDHARS NATGCAQMPT NEQATCWQAV 98
AFR-23270 MPAKILFLLL VLALSGCAS- -LQPPS-STA TASAAARSVA MANRDAEAAQ QRLAAVAAQR AGAEQQFCPN WRQALGQARR NAMGCARMPL GEQATCWQAV 97
AFR-53993 MPAKILFLLL VLALSGCAS- -LQPPS-STA TASAAARSVA MANRDAEAAQ QRLAAVAAQR AGAEQQFCPN WRQALGQARR NAMGCARMPL GEQATCWQAV 97
ATH-19377 MPAKILLLLV LASLAGCAT- -ITPSGPNHL TSSAATQSAQ LAQQKAELAE RHLAAIAGQR ATAERQFCPN WQQALLHARN NAIGCAQIPI NAQSACWQAV 98
ATH-LCY MPAKILLLLV LASLAGCAT- -ITPSGPNHL TSSAATQSAQ LAQQKAELAE RHLAAIAGQR ATAERQFCPN WQQALLHARN NAIGCAQMPI NAQSACWQAV 98
ATH-A01 MPAKILLLLV LASLAGCAT- -VTPSGPNQQ TSSAATQSAR LAQQKAELAE QHLAAIASQR ATAERQFCPN WQQALLHARN NAIGCARMPI NAQSACWQAV 98
ACA-SM1 MRKWFFSGLV ILGLGGCASN PMMPPSPTGA AATAEARSQA AA-RAAQEAQ QKLAATAVQR RAAEGQFCAS WRRALDLARR DAIGCARMEA DQQAACWSAV 99
ACA-51756 MRKWFFSGLV ILGLGGCASN PMMPPSPTGA AATAEARSQA AA-RAAQEAQ QKLAATAVQR RAAEGQFCAS WRRALDLARR DAIGCARMEA DQQAACWSAV 99
Consensus MPAKILFLLV LLALSGCAS- -LTPSSPSTA TASAAARSAA LANRKAELAQ QRLAAVAAQR ATAEQQFCPN WQQALDHARR NAIGCARMPI NEQAACWQAV
Conservation
4.3bits
Sequence logo
0.0bits
120 140 160
AFV-SS3 SQWAQEESHY FHALAPLLQR SAYASPAAQA AHFFDLTQGW AITCQNGQKA CTAASGHQQM DNSKNAINQF C-RR 171
AFV-CF27 SQWAQEESRY FHALAPLLQR SAYASPAAQA AHFFDLTQGW AITCQNGQKA CTAASGHQQM DNSKNAINHF C-RR 171
AFR-23270 SQWTQEESRY FHALAPLFQE GAYATPAAQA ARFFDLAQGW AITCQDGQKA CSAASGHQQM DDHKNVVNRF C-SR 170
AFR-53993 SQWTQEESRY FHALAPLFQE GAYATPAAQA ARFFDLAQGW AITCQDGQKA CSAASGHQQM DDHKNVVNRF C-SR 170
ATH-19377 AQWTNEESQY FHALHPLFTH SPYAEPAGHA AHFFDLAQSW AMTCEDGGAA CTQASGHQQM DQEKKQVNQF CMHQ 172
ATH-LCY AQWTNEESQY FHALHPLFTH SPYAEPAGHA AHFFDLAQSW AMTCEDGGAA CTQASGHQQM DQEKKQVNQF CMHQ 172
ATH-A01 TQWANEESQY FHALHPLFTH SPYAEPAGHA AHFFDLAQSW AMTCEDGGAA CTQASGHQQM DQEKKQVNQF CMHQ 172
ACA-SM1 AQWAGEESRY FSALESLFSE GPYATSAGKA GEFFHLTQSW ATTCGDSLAD CTSAPQRATM DQRKLEVNRF C--H 171
ACA-51756 AQWAGEESRY FSALESLFSE GPYATSAGKA GEFFHLTQSW ATTCGDSLAD CTSAPQRATM DQRKLEVNRF C--H 171
Consensus XQWAQEESRY FHALAPLFQE SPYATPAGQA AHFFDLAQSW AITCQDGQAA CTAASGHQQM DQEKNQVNXF C-HR
Conservation
4.3bits
Sequence logo
0.0bits

Family V

```
                                                        20                                      40                                      60                                      80                                      100
  AFV-SS3     MQQPAIYSAA  QSVFHSS-KR  GFVGSALFAL  GLFGLGTSVA  FASGMETSAP  GLLRTAMTAG  AVASPAEIKN  FAAAVRGIKP  LNEQVHSALS  QKGITATKRE   99
  AFV-CF27    MQQPAIYSAA  QSVFHSS-KR  GFVGSALFAF  GLLGLGTSVA  FASSMEMSAP  GLVRTAMTAG  AVASPAEIKN  FAAAVRGIKP  LNEQVHSALS  QKGITATKRE   99
  AFR-23270   MQKLPVYSAA  QGLFHSPLRR  GMAGVVLAAF  GLMGLGAGTA  FASSIGTSAP  GLMRTAVAGG  AAASPAEVKN  FAAAVQEIKP  LNEQVHTALS  RKGITATQRE  100
  AFR-53993   MQKLPVYSAA  QGLFHSPLRR  GMAGVVLAAF  GLMGLGAGTA  FASSIGTSAP  GLMRTAVAGG  AAASPAEVKN  FAAAVQEIKP  LNEQVHTALS  RKGITATQRE  100
  ATH-A01     MQK-------  -SLGHSIK--  --FGLVVLAL  GGLGLVSNVA  FASD------  ---------A  MAASPAQIKD  FAHAVAQIKP  LNVQAHSEIS  QKGVSKAKQD   73
  ATH-LCY     MQK-------  -SSSHSLK--  --FGLVVLAL  GGLGLGSNVA  FASD------  ---------A  MAASPAQIKD  FAHAVAQIKP  LNAQAHAEIS  QKGVSKAKQD   73
  ATH-19377   MQK-------  -SLGHSIK--  --FGLVVLAL  GGLGLVSNVA  FASD------  ---------A  MAASPAQIKD  FAHAVEQIKP  LNAQAHAEIS  QKGVSKAKQD   73
  ACA-SM1     MKHRISTQHS  QRAAHGTSR-  --IGGLILAA  GTCLLLANGA  FAAP------  ---------T  PQLSPAQLHD  FAGAVKELQG  LNNQVHQEAM  NPKLTPAQKE   82
  ACA-51756   MKHRISTQHS  QRAAHGTSR-  --IGGLILAA  GTCLLLANGA  FAAP------  ---------T  PQLSPAQLHD  FAGAVKELQG  LNNQVHQEAM  NPKLTPAQKE   82
  Consensus   MQK---YSAA  QSLFHSXKR-  --FGLVLLAL  GLLGLGANVA  FASX------  ---------G  AAASPAQIKD  FAAAVXEIKP  LNEQVHSELS  QKGITAAKRE
Conservation
4.3bits
Sequence logo
0.0bits

                                                        120                                     140
  AFV-SS3     ELKKSYMARV  NSILASNHLT  AEQYTSMLKQ  TQDDPAFARQ  VEGAMQ  145
  AFV-CF27    ELKKSYMARV  NSILASNHLT  AEQYTSMLKQ  TQDDPAFAKQ  VEGAMQ  145
  AFR-23270   ELKKSYMTKV  DDILAKNHLT  AEQYSSLLRQ  TQSDPDFAKE  VESDMR  146
  AFR-53993   ELKKSYMTKV  DDILAKNHLT  AEQYSSLLRQ  TQSDPDFAKE  VESDMR  146
  ATH-A01     AVKKSYMEKV  DKVLATNHLS  AGQYSSMLKQ  TQTDPAFAKQ  VEAAMH  119
  ATH-LCY     AVKKSYVEKL  DKVLATNHLS  AEQYSSMLKQ  TQTDPAFLKQ  VEAAMH  119
  ATH-19377   AVKNPIIPFI  N---------  ----------  ----------  ------   84
  ACA-SM1     KLKNEYMAKT  NAILAQHHLT  AQRYTELLQE  TQRDPAFAKE  VEGAMH  128
  ACA-51756   KLKNEYMAKT  NAILAQHHLT  AQRYTELLQE  TQRDPAFAKE  VEGAMH  128
  Consensus   ELKKSYMAKV  NXILAXNHLT  AEQYXSXLKQ  TQXDPAFAKX  VEGAMH
Conservation
4.3bits
Sequence logo
0.0bits
```

Family I

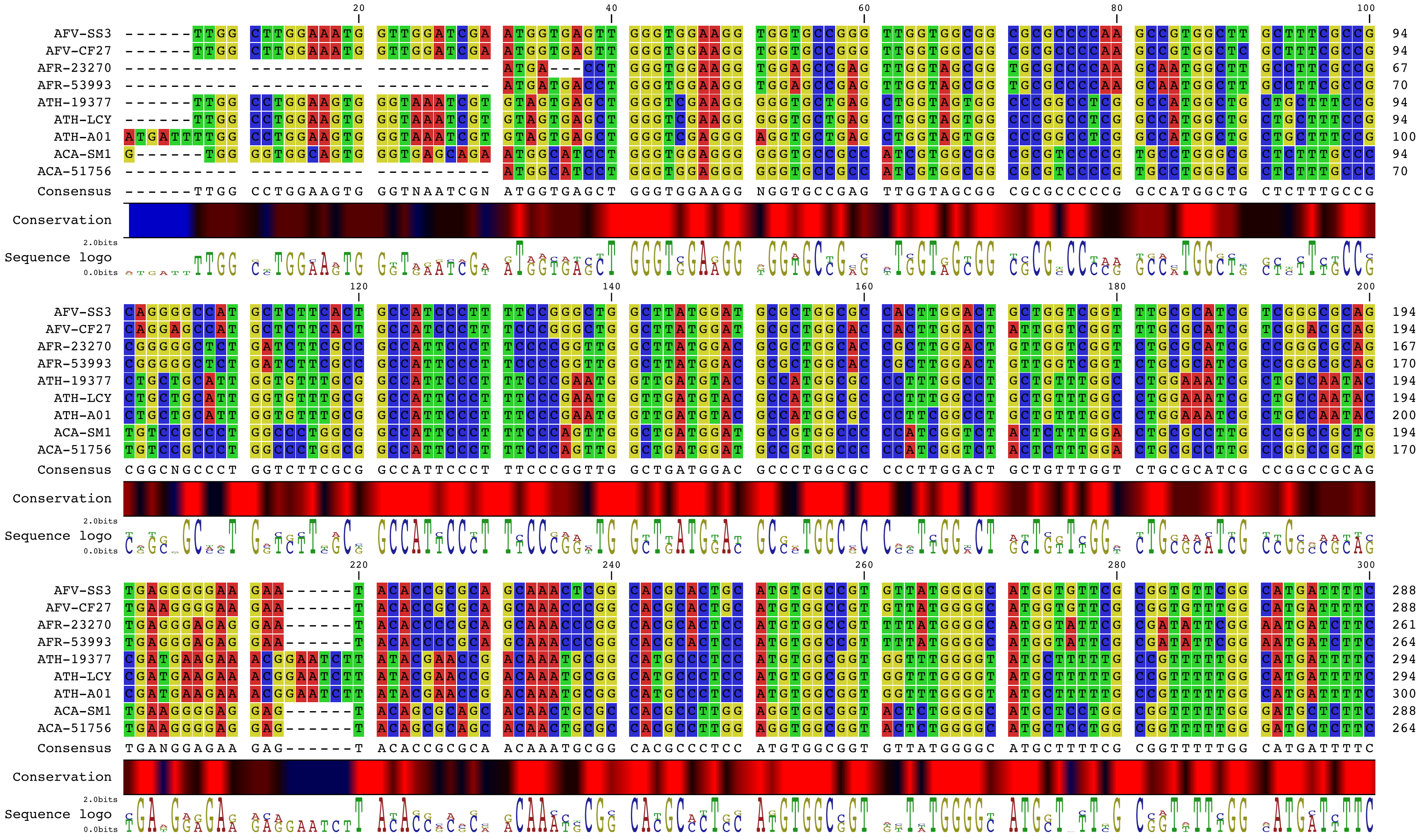

AFV-SS3
AFV-CF27
AFR-23270
AFR-53993
ATH-19377
ATH-LCY
ATH-A01
ACA-SM1
ACA-51756
Consensus
Conservation
Sequence logo
2.0bits
0.0bits

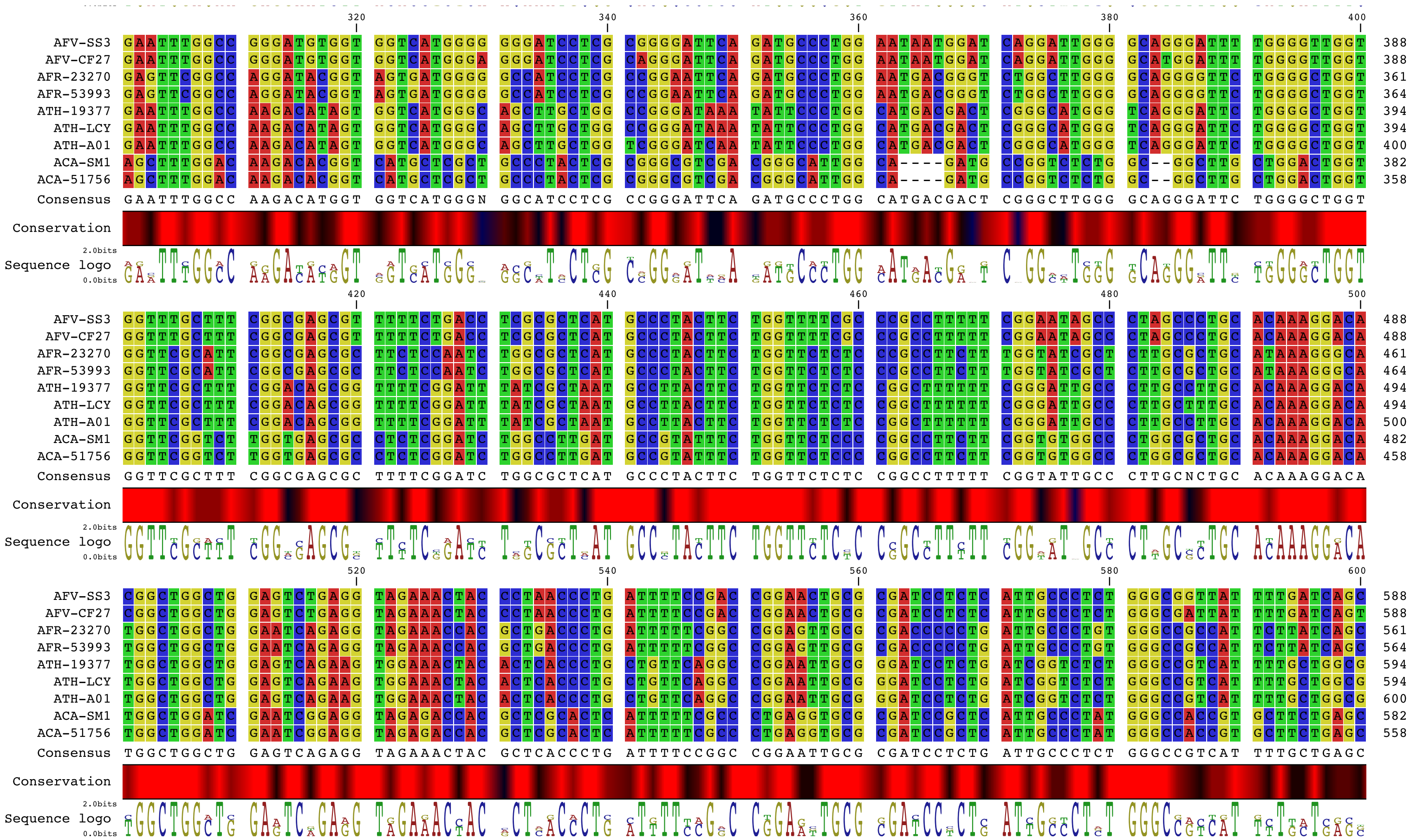

320 340 360 380 400
AFV-SS3 388
AFV-CF27 388
AFR-23270 361
AFR-53993 364
ATH-19377 394
ATH-LCY 394
ATH-A01 400
ACA-SM1 382
ACA-51756 358
Consensus GAATTTGGCC AAGACATGGT GGTCATGGGN GGCATCCTCG CCGGGATTCA GATGCCCTGG CATGACGACT CGGGCTTGGG GCAGGGATTC TGGGGCTGGT
Conservation
Sequence logo
2.0bits
0.0bits
420 440 460 480 500
AFV-SS3 488
AFV-CF27 488
AFR-23270 461
AFR-53993 464
ATH-19377 494
ATH-LCY 494
ATH-A01 500
ACA-SM1 482
ACA-51756 458
Consensus GGTTCGCTTT CGGCGAGCGC TTTTCGGATC TGGCGCTCAT GCCCTACTTC TGGTTCTCTC CGGCCTTTTT CGGTATTGCC CTTGCNCTGC ACAAAGGACA
Conservation
Sequence logo
520 540 560 580 600
AFV-SS3 588
AFV-CF27 588
AFR-23270 561
AFR-53993 564
ATH-19377 594
ATH-LCY 594
ATH-A01 600
ACA-SM1 582
ACA-51756 558
Consensus TGGCTGGCTG GAGTCAGAGG TAGAAACTAC GCTCACCCTG ATTTTCCGGC CGGAATTGCG CGATCCTCTG ATTGCCCTCT GGGCCGTCAT TTTGCTGAGC
Conservation
Sequence logo

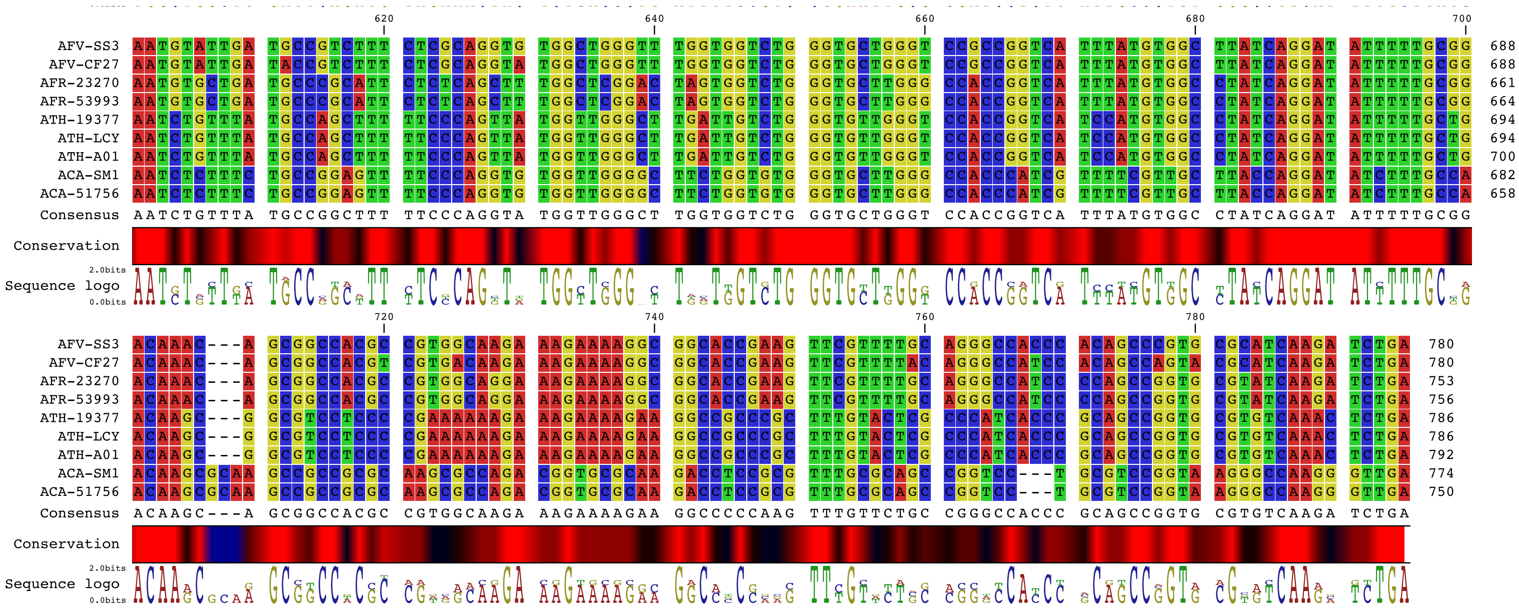
620 640 660 680 700
AFV-SS3 AATGTATTGA TGCCGTCTTT CTCGCAGGTG TGGCTGGGTT TGGTGGTCTG GGTGCTGGGT CCGCCGGTCA TTTATGTGGC TTATCAGGAT ATTTTTGCGG 688
AFV-CF27 AATGTATTGA TACCGTCTTT CTCGCAGGTA TGGCTGGGTT TGGTGGTCTG GGTGCTGGGT CCGCCGGTCA TTTATGTGGC TTATCAGGAT ATTTTTGCGG 688
AFR-23270 AATGTGCTGA TGCCCGCATT CTCTCAGCTT TGGCTCGGAC TAGTGGTCTG GGTGCTTGGG CCACCGGTCA TTTATGTGGC CTATCAGGAT ATTTTTGCGG 661
AFR-53993 AATGTGCTGA TGCCCGCATT CTCTCAGCTT TGGCTCGGAC TAGTGGTCTG GGTGCTTGGG CCACCGGTCA TTTATGTGGC CTATCAGGAT ATTTTTGCGG 664
ATH-19377 AATCTGTTTA TGCCAGCTTT TTCCCAGTTA TGGTTGGGCT TGATTGTCTG GGTGTTGGGT CCACCGGTCA TCCATGTGGC CTATCAGGAT ATTTTTGCTG 694
ATH-LCY AATCTGTTTA TGCCAGCTTT TTCCCAGTTA TGGTTGGGCT TGATTGTCTG GGTGTTGGGT CCACCGGTCA TCCATGTGGC CTATCAGGAT ATTTTTGCTG 694
ATH-A01 AATCTGTTTA TGCCAGCTTT TTCCCAGTTA TGGTTGGGCT TGATTGTCTG GGTGTTGGGT CCACCGGTCA TCCATGTGGC CTATCAGGAT ATTTTTGCTG 700
ACA-SM1 AATCTCTTTC TGCCGGAGTT TTCCCAGGTG TGGTTGGGGC TTCTGGTGTG GGTGCTTGGG CCACCCATCG TTTTCGTTGC TTACCAGGAT ATCTTTGCCA 682
ACA-51756 AATCTCTTTC TGCCGGAGTT TTCCCAGGTG TGGTTGGGGC TTCTGGTGTG GGTGCTTGGG CCACCCATCG TTTTCGTTGC TTACCAGGAT ATCTTTGCCA 658
Consensus AATCTGTTTA TGCCGGCTTT TTCCCAGGTA TGGTTGGGCT TGGTGGTCTG GGTGCTGGGT CCACCGGTCA TTTATGTGGC CTATCAGGAT ATTTTTGCGG
Conservation
2.0bits
Sequence logo
0.0bits
720 740 760 780
AFV-SS3 ACAAAC---A GCGGCCACGC CGTGGCAAGA AAGAAAAGGC GGCACCGAAG TTCGTTTTGC AGGGCCACCC ACAGCCCGTG CGCATCAAGA TCTGA 780
AFV-CF27 ACAAAC---A GCGGCCACGT CGTGACAAGA AAGAAAAGGC GGCACCGAAG TTCGTTTTAC AGGGCCATCC ACAGCCAGTA CGCATCAAGA TCTGA 780
AFR-23270 ACAAAC---A GCGGCCACGC CGTGGCAGGA AAGAAAAGGC GGCACCGAAG TTCGTTTTGC AGGGCCATCC CCAGCCGGTG CGTATCAAGA TCTGA 753
AFR-53993 ACAAAC---A GCGGCCACGC CGTGGCAGGA AAGAAAAGGC GGCACCGAAG TTCGTTTTGC AGGGCCATCC CCAGCCGGTG CGTATCAAGA TCTGA 756
ATH-19377 ACAAGC---G GCGTCCTCCC CGAAAAAAGA AAGAAAAGAA GGCCGCCCGC TTTGTACTCG CCCATCACCC GCAGCCGGTG CGTGTCAAAC TCTGA 786
ATH-LCY ACAAGC---G GCGTCCTCCC CGAAAAAAGA AAGAAAAGAA GGCCGCCCGC TTTGTACTCG CCCATCACCC GCAGCCGGTG CGTGTCAAAC TCTGA 786
ATH-A01 ACAAGC---G GCGTCCTCCC CGAAAAAAGA AAGAAAAGAA GGCCGCCCGC TTTGTACTCG CCCATCACCC GCAGCCGGTG CGTGTCAAAC TCTGA 792
ACA-SM1 ACAAGCGCAA GCCGCCGCGC AAGCGCCAGA CGGTGCGCAA GACCTCCGCG TTTGCGCAGC CGGTCC---T GCGTCCGGTA AGGGCCAAGG GTTGA 774
ACA-51756 ACAAGCGCAA GCCGCCGCGC AAGCGCCAGA CGGTGCGCAA GACCTCCGCG TTTGCGCAGC CGGTCC---T GCGTCCGGTA AGGGCCAAGG GTTGA 750
Consensus ACAAGC---A GCGGCCACGC CGTGGCAAGA AAGAAAAGAA GGCCCCCAAG TTTGTTCTGC CGGGCCACCC GCAGCCGGTG CGTGTCAAGA TCTGA
Conservation
2.0bits
Sequence logo
0.0bits

Family II

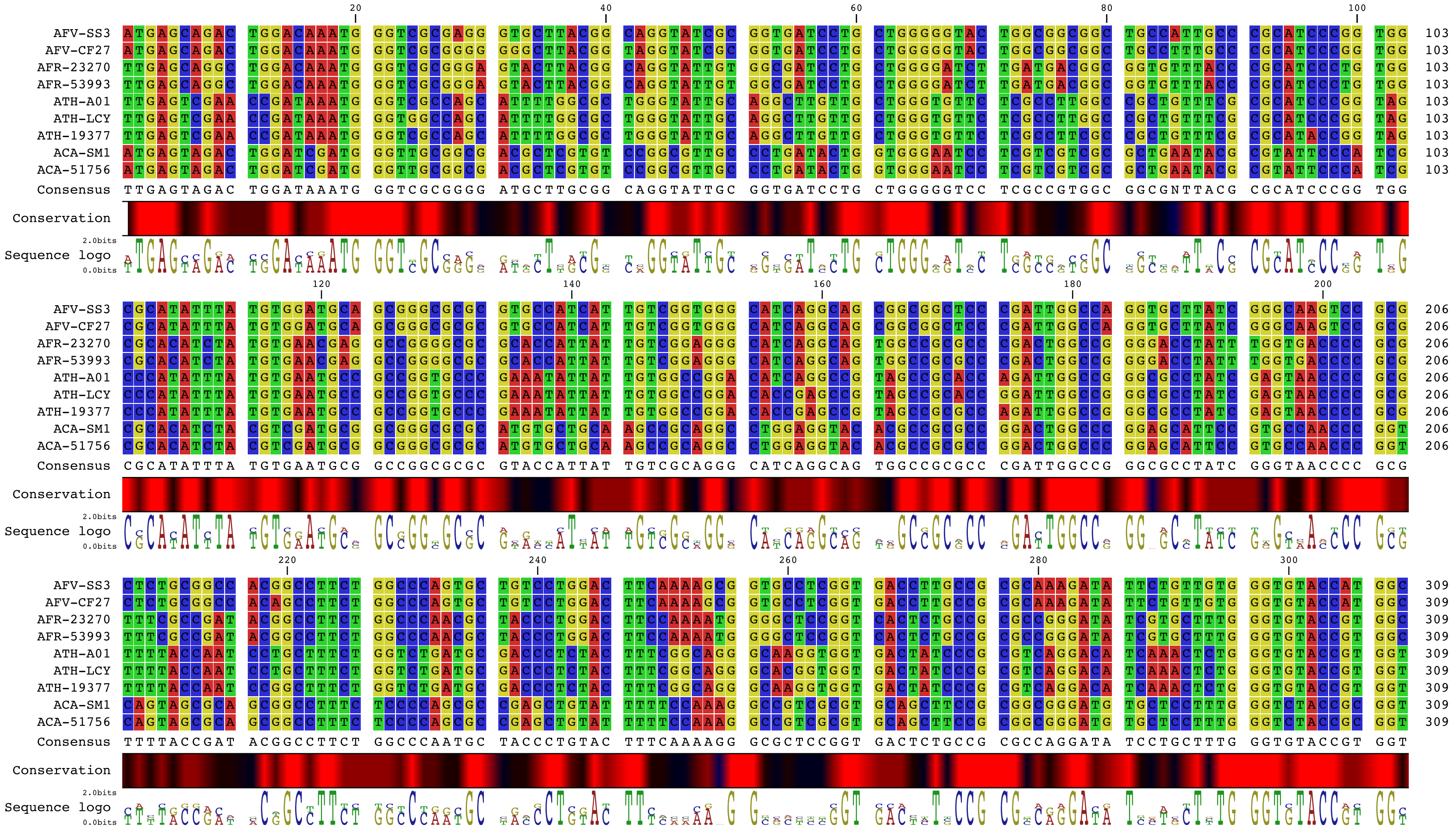
AFV-SS3
AFV-CF27
AFR-23270
AFR-53993
ATH-A01
ATH-LCY
ATH-19377
ACA-SM1
ACA-51756
Consensus
Conservation
Sequence logo

AFV-SS3	T	G	A	312
AFV-CF27	T	G	A	312
AFR-23270	T	G	A	312
AFR-53993	T	G	A	312
ATH-A01	T	A	A	312
ATH-LCY	T	A	A	312
ATH-19377	T	A	A	312
ACA-SM1	T	G	A	312
ACA-51756	T	G	A	312
Consensus	T	G	A	

Conservation

Sequence logo

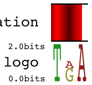

Family III

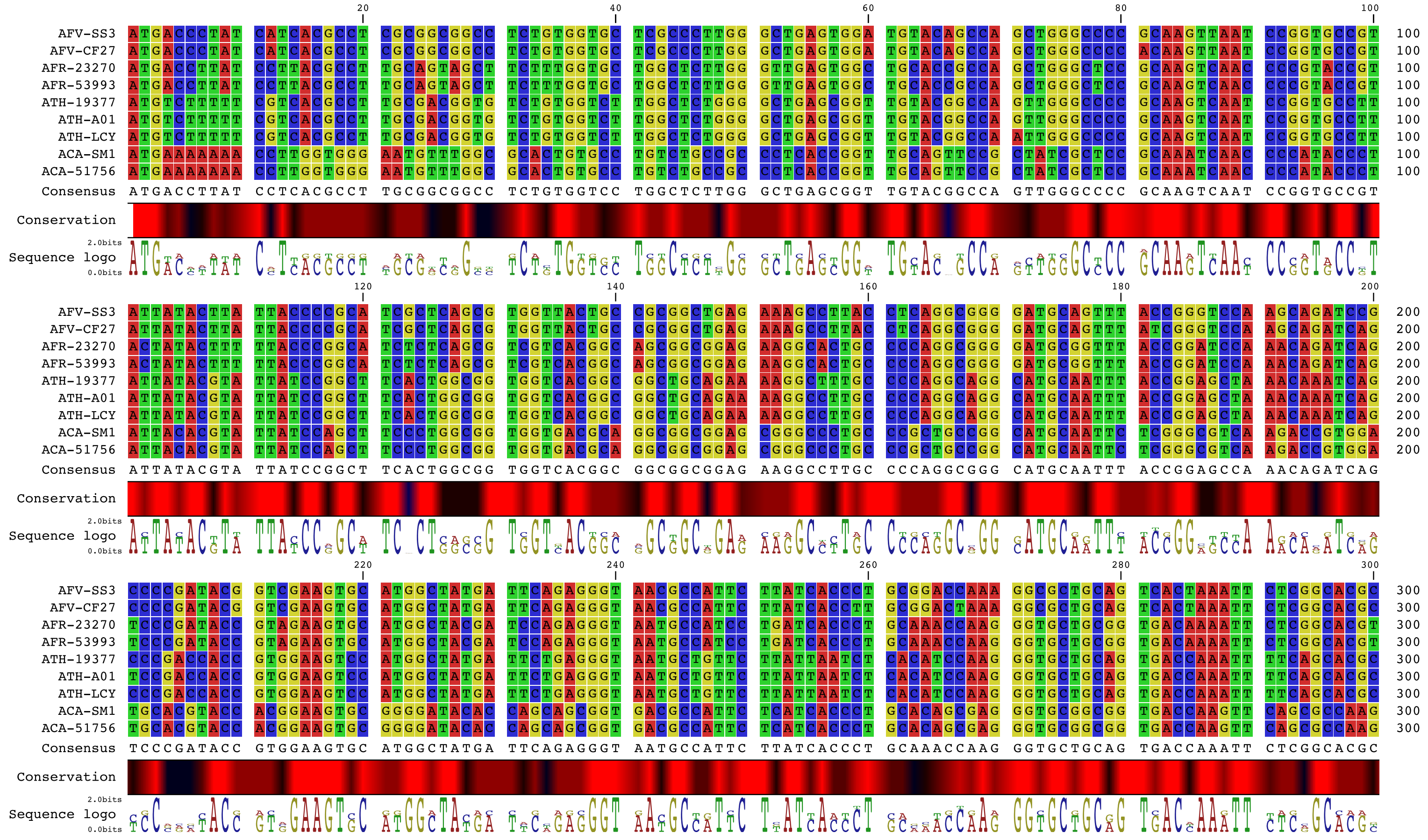
AFV-SS3
AFV-CF27
AFR-23270
AFR-53993
ATH-19377
ATH-A01
ATH-LCY
ACA-SM1
ACA-51756
Consensus
Conservation
Sequence logo

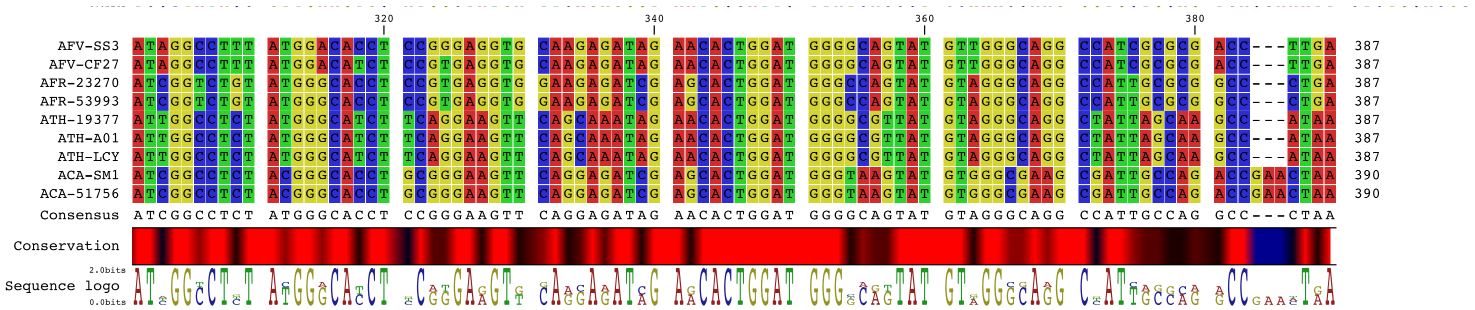

320
340
360
380
AFV-SS3 ATAGGCCTTT ATGGACACCT CCGGGAGGTG CAAGAGATAG AACACTGGAT GGGGCAGTAT GTTGGGCAGG CCATCGCGCG ACC---TTGA 387
AFV-CF27 ATAGGCCTTT ATGGACATCT CCGTGAGGTG CAAGAGATAG AACACTGGAT GGGGCAGTAT GTTGGGCAGG CCATCGCGCG ACC---TTGA 387
AFR-23270 ATCGGTCTGT ATGGGCACCT CCGTGAGGTG GAAGAGATCG AGCACTGGAT GGGCCAGTAT GTAGGGCAGG CCATTGCGCG GCC---CTGA 387
AFR-53993 ATCGGTCTGT ATGGGCACCT CCGTGAGGTG GAAGAGATCG AGCACTGGAT GGGCCAGTAT GTAGGGCAGG CCATTGCGCG GCC---CTGA 387
ATH-19377 ATTGGCCTCT ATGGGCATCT TCAGGAAGTT CAGCAAATAG AACACTGGAT GGGGCGTTAT GTAGGGCAGG CTATTAGCAA GCC---ATAA 387
ATH-A01 ATTGGCCTCT ATGGGCATCT TCAGGAAGTT CAGCAAATAG AACACTGGAT GGGGCGTTAT GTAGGGCAGG CTATTAGCAA GCC---ATAA 387
ATH-LCY ATTGGCCTCT ATGGGCATCT TCAGGAAGTT CAGCAAATAG AACACTGGAT GGGGCGTTAT GTAGGGCAGG CTATTAGCAA GCC---ATAA 387
ACA-SM1 ATCGGCCTCT ACGGGCACCT GCGGGAAGTT CAGGAGATCG AGCACTGGAT GGGTAAGTAT GTGGGCGAAG CGATTGCCAG ACCGAACTAA 390
ACA-51756 ATCGGCCTCT ACGGGCACCT GCGGGAAGTT CAGGAGATCG AGCACTGGAT GGGTAAGTAT GTGGGCGAAG CGATTGCCAG ACCGAACTAA 390
Consensus ATCGGCCTCT ATGGGCACCT CCGGGAAGTT CAGGAGATAG AACACTGGAT GGGGCAGTAT GTAGGGCAGG CCATTGCCAG GCC---CTAA
Conservation
2.0bits
Sequence logo
0.0bits

Family IV

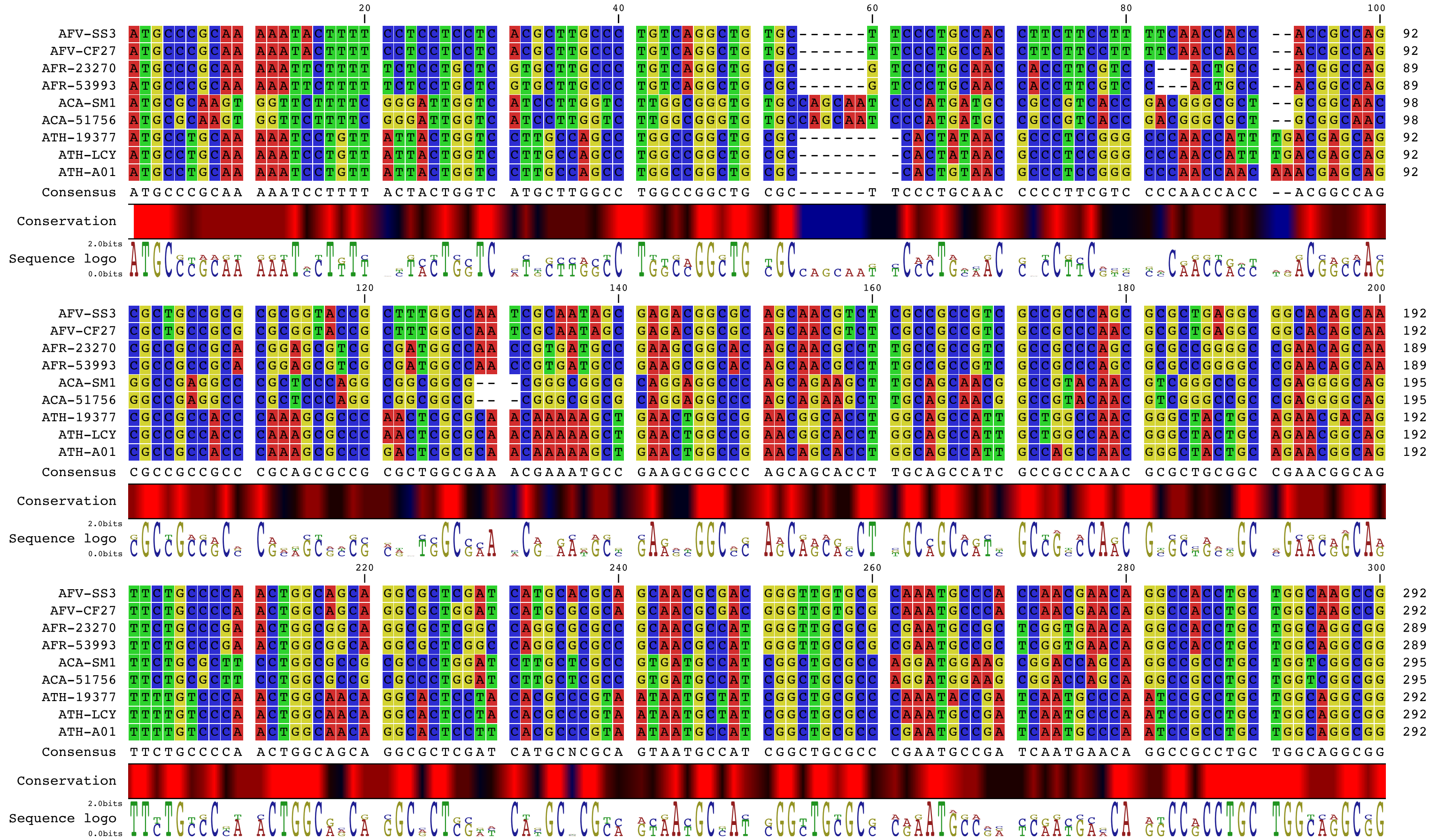

20
40
60
80
100
120
140
160
180
200
220
240
260
280
300
AFV-SS3 92 192 292
AFV-CF27 92 192 292
AFR-23270 89 189 289
AFR-53993 89 189 289
ACA-SM1 98 195 295
ACA-51756 98 195 295
ATH-19377 92 192 292
ATH-LCY 92 192 292
ATH-A01 92 192 292
Consensus ATGCCCGCAA AAATCCTTTT ACTACTGGTC ATGCTTGGCC TGGCCGGCTG CGC------T TCCCTGCAAC CCCCTTCGTC CCCAACCACC --ACGGCCAG
Consensus CGCCGCCGCC CGCAGCGCCG CGCTGGCGAA ACGAAATGCC GAAGCGGCCC AGCAGCACCT TGCAGCCATC GCCGCCCAAC GCGCTGCGGC CGAACGGCAG
Consensus TTCTGCCCCA ACTGGCAGCA GGCGCTCGAT CATGCNCGCA GTAATGCCAT CGGCTGCGCC CGAATGCCGA TCAATGAACA GGCCGCCTGC TGGCAGGCGG
Conservation
Sequence logo
2.0bits
0.0bits

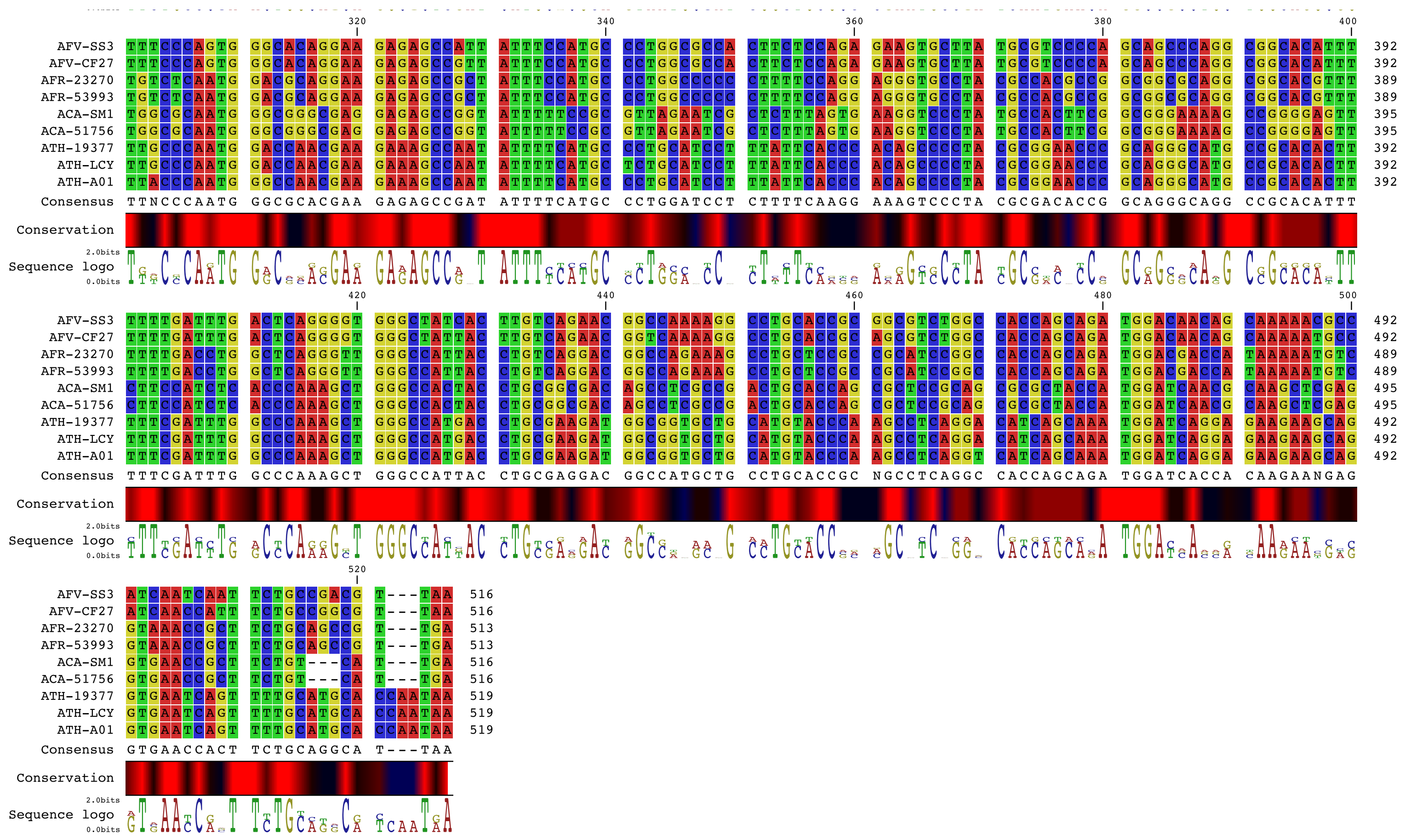

320 340 360 380 400
AFV-SS3 TTTCCCAGTG GGCACAGGAA GAGAGCCATT ATTTCCATGC CCTGGCGCCA CTTCTCCAGA GAAGTGCTTA TGCGTCCCCA GCAGCCCAGG CGGCACATTT 392
AFV-CF27 TTTCCCAGTG GGCACAGGAA GAGAGCCGTT ATTTCCATGC CCTGGCGCCA CTTCTCCAGA GAAGTGCTTA TGCGTCCCCA GCAGCCCAGG CGGCACATTT 392
AFR-23270 TGTCTCAATG GACGCAGGAA GAGAGCCGCT ATTTCCATGC CCTGGCCCCC CTTTTCCAGG AGGGTGCCTA CGCCACGCCG GCGGCGCAGG CGGCACGTTT 389
AFR-53993 TGTCTCAATG GACGCAGGAA GAGAGCCGCT ATTTCCATGC CCTGGCCCCC CTTTTCCAGG AGGGTGCCTA CGCCACGCCG GCGGCGCAGG CGGCACGTTT 389
ACA-SM1 TGGCGCAATG GGCGGGCGAG GAGAGCCGGT ATTTTTCCGC GTTAGAATCG CTCTTTAGTG AAGGTCCCTA TGCCACTTCG GCGGGAAAAG CCGGGGAGTT 395
ACA-51756 TGGCGCAATG GGCGGGCGAG GAGAGCCGGT ATTTTTCCGC GTTAGAATCG CTCTTTAGTG AAGGTCCCTA TGCCACTTCG GCGGGAAAAG CCGGGGAGTT 395
ATH-19377 TTGCCCAATG GACCAACGAA GAAAGCCAAT ATTTTCATGC CCTGCATCCT TTATTCACCC ACAGCCCCTA CGCGGAACCC GCAGGGCATG CCGCACACTT 392
ATH-LCY TTGCCCAATG GACCAACGAA GAAAGCCAAT ATTTTCATGC TCTGCATCCT TTATTCACCC ACAGCCCCTA CGCGGAACCC GCAGGGCATG CCGCACACTT 392
ATH-A01 TTACCCAATG GGCCAACGAA GAAAGCCAAT ATTTTCATGC CCTGCATCCT TTATTCACCC ACAGCCCCTA CGCGGAACCC GCAGGGCATG CCGCACACTT 392
Consensus TTNCCCAATG GGCGCACGAA GAGAGCCGAT ATTTTCATGC CCTGGATCCT CTTTTCAAGG AAAGTCCCTA CGCGACACCG GCAGGGCAGG CCGCACATTT
Conservation
2.0bits
Sequence logo
0.0bits
420 440 460 480 500
AFV-SS3 TTTTGATTTG ACTCAGGGGT GGGCTATCAC TTGTCAGAAC GGCCAAAAGG CCTGCACCGC GGCGTCTGGC CACCAGCAGA TGGACAACAG CAAAAACGCC 492
AFV-CF27 TTTTGATTTG ACTCAGGGGT GGGCTATTAC TTGTCAGAAC GGTCAAAAGG CCTGCACCGC AGCGTCTGGC CACCAGCAGA TGGACAACAG CAAAAATGCC 492
AFR-23270 TTTTGACCTG GCTCAGGGTT GGGCCATTAC CTGTCAGGAC GGCCAGAAAG CCTGCTCCGC CGCATCCGGC CACCAGCAGA TGGACGACCA TAAAAATGTC 489
AFR-53993 TTTTGACCTG GCTCAGGGTT GGGCCATTAC CTGTCAGGAC GGCCAGAAAG CCTGCTCCGC CGCATCCGGC CACCAGCAGA TGGACGACCA TAAAAATGTC 489
ACA-SM1 CTTCCATCTC ACCCAAAGCT GGGCCACTAC CTGCGGCGAC AGCCTCGCCG ACTGCACCAG CGCTCCGCAG GGCGCTACCA TGGATCAACG CAAGCTCGAG 495
ACA-51756 CTTCCATCTC ACCCAAAGCT GGGCCACTAC CTGCGGCGAC AGCCTCGCCG ACTGCACCAG CGCTCCGCAG GGCGCTACCA TGGATCAACG CAAGCTCGAG 495
ATH-19377 TTTCGATTTG GCCCAAAGCT GGGCCATGAC CTGCGAAGAT GGCGGTGCTG CATGTACCCA AGCCTCAGGA CATCAGCAAA TGGATCAGGA GAAGAAGCAG 492
ATH-LCY TTTCGATTTG GCCCAAAGCT GGGCCATGAC CTGCGAAGAT GGCGGTGCTG CATGTACCCA AGCCTCAGGA CATCAGCAAA TGGATCAGGA GAAGAAGCAG 492
ATH-A01 TTTCGATTTG GCCCAAAGCT GGGCCATGAC CTGCGAAGAT GGCGGTGCTG CATGTACCCA AGCCTCAGGT CATCAGCAAA TGGATCAGGA GAAGAAGCAG 492
Consensus TTTCGATTTG GCCCAAAGCT GGGCCATTAC CTGCGAGGAC GGCCATGCTG CCTGCACCGC NGCCTCAGGC CACCAGCAGA TGGATCACCA CAAGAANGAG
Conservation
2.0bits
Sequence logo
0.0bits
520
AFV-SS3 ATCAATCAAT TCTGCCGACG T---TAA 516
AFV-CF27 ATCAACCATT TCTGCCGGCG T---TAA 516
AFR-23270 GTAAACCGCT TCTGCAGCCG T---TGA 513
AFR-53993 GTAAACCGCT TCTGCAGCCG T---TGA 513
ACA-SM1 GTGAACCGCT TCTGT---CA T---TGA 516
ACA-51756 GTGAACCGCT TCTGT---CA T---TGA 516
ATH-19377 GTGAATCAGT TTTGCATGCA CCAATAA 519
ATH-LCY GTGAATCAGT TTTGCATGCA CCAATAA 519
ATH-A01 GTGAATCAGT TTTGCATGCA CCAATAA 519
Consensus GTGAACCACT TCTGCAGGCA T---TAA
Conservation
2.0bits
Sequence logo
0.0bits

Family V

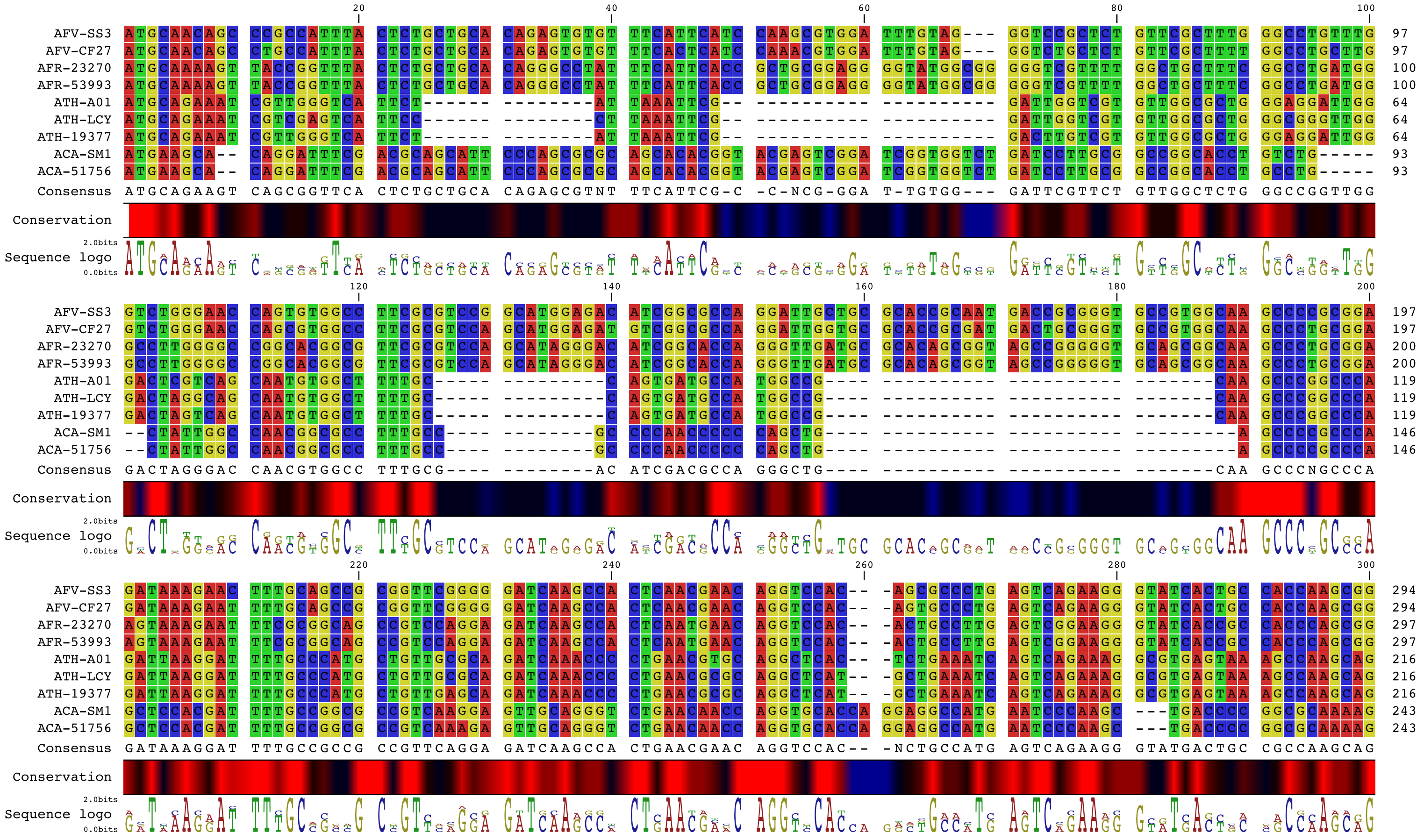

20 40 60 80 100
AFV-SS3 ATGCAACAGC CCGCCATTTA CTCTGCTGCA CAGAGTGTGT TTCATTCATC CAAGCGTGGA TTTGTAG--- GGTCCGCTCT GTTCGCTTTG GGCCTGTTTG 97
AFV-CF27 ATGCAACAGC CTGCCATTTA CTCTGCTGCA CAGAGTGTGT TTCACTCATC CAAACGTGGA TTTGTAG--- GGTCTGCTCT GTTCGCTTTT GGCCTGCTTG 97
AFR-23270 ATGCAAAAGT TACCGGTTTA CTCTGCTGCA CAGGGCCTAT TTCATTCACC GCTGCGGAGG GGTATGGCGG GGGTCGTTTT GGCTGCTTTC GGCCTGATGG 100
AFR-53993 ATGCAAAAGT TACCGGTTTA CTCTGCTGCA CAGGGCCTAT TTCATTCACC GCTGCGGAGG GGTATGGCGG GGGTCGTTTT GGCTGCTTTC GGCCTGATGG 100
ATH-A01 ATGCAGAAAT CGTTGGGTCA TTCT------ --------AT TAAATTCG-- ---------- ---------- GATTGGTCGT GTTGGCGCTG GGAGGATTGG 64
ATH-LCY ATGCAGAAAT CGTCGAGTCA TTCC------ --------CT TAAATTCG-- ---------- ---------- GATTGGTCGT GTTGGCGCTG GGCGGGTTGG 64
ATH-19377 ATGCAGAAAT CGTTGGGTCA TTCT------ --------AT TAAATTCG-- ---------- ---------- GACTTGTCGT GTTGGCGCTG GGAGGATTGG 64
ACA-SM1 ATGAAGCA-- CAGGATTTCG ACGCAGCATT CCCAGCGCGC AGCACACGGT ACGAGTCGGA TCGGTGGTCT GATCCTTGCG GCCGGCACCT GTCTG----- 93
ACA-51756 ATGAAGCA-- CAGGATTTCG ACGCAGCATT CCCAGCGCGC AGCACACGGT ACGAGTCGGA TCGGTGGTCT GATCCTTGCG GCCGGCACCT GCCTG----- 93
Consensus ATGCAGAAGT CAGCGGTTCA CTCTGCTGCA CAGAGCGTNT TTCATTCG-C -C-NCG-GGA T-TGTGG--- GATTCGTTCT GTTGGCTCTG GGCCGGTTGG
Conservation
2.0bits
Sequence logo
0.0bits
120 140 160 180 200
AFV-SS3 GTCTGGGAAC CAGTGTGGCC TTCGCGTCCG GCATGGAGAC ATCGGCGCCA GGATTGCTGC GCACCGCAAT GACCGCGGGT GCCGTGGCAA GCCCCGCGGA 197
AFV-CF27 GTCTGGGAAC CAGCGTGGCC TTCGCGTCCA GCATGGAGAT GTCGGCGCCA GGATTGGTGC GCACCGCGAT GACTGCGGGT GCCGTGGCAA GCCCTGCGGA 197
AFR-23270 GCCTTGGGGC CGGCACGGCG TTCGCGTCCA GCATAGGGAC ATCGGCACCA GGGTTGATGC GCACAGCGGT AGCCGGGGGT GCAGCGGCAA GCCCTGCGGA 200
AFR-53993 GCCTTGGGGC CGGCACGGCG TTCGCGTCCA GCATAGGGAC ATCGGCACCA GGGTTGATGC GCACAGCGGT AGCCGGGGGT GCAGCGGCAA GCCCTGCGGA 200
ATH-A01 GACTCGTCAG CAATGTGGCT TTTGC----- ---------C AGTGATGCCA TGGCCG---- ---------- ---------- -------CAA GCCCGGCCCA 119
ATH-LCY GACTAGGCAG CAATGTGGCT TTTGC----- ---------C AGTGATGCCA TGGCCG---- ---------- ---------- -------CAA GCCCGGCCCA 119
ATH-19377 GACTAGTCAG CAATGTGGCT TTTGC----- ---------C AGTGATGCCA TGGCCG---- ---------- ---------- -------CAA GCCCGGCCCA 119
ACA-SM1 --CTATTGGC CAACGGCGCC TTTGCC---- --------GC CCCAACCCCC CAGCTG---- ---------- ---------- ---------A GCCCCGCCCA 146
ACA-51756 --CTATTGGC CAACGGCGCC TTTGCC---- --------GC CCCAACCCCC CAGCTG---- ---------- ---------- ---------A GCCCCGCCCA 146
Consensus GACTAGGGAC CAACGTGGCC TTTGCG---- --------AC ATCGACGCCA GGGCTG---- ---------- ---------- -------CAA GCCCNGCCCA
Conservation
2.0bits
Sequence logo
0.0bits
220 240 260 280 300
AFV-SS3 GATAAAGAAC TTTGCAGCCG CGGTTCGGGG GATCAAGCCA CTCAACGAAC AGGTCCAC-- -AGCGCCCTG AGTCAGAAGG GTATCACTGC CACCAAGCGG 294
AFV-CF27 GATAAAGAAT TTTGCAGCCG CGGTTCGGGG GATCAAGCCA CTCAACGAAC AGGTCCAC-- -AGTGCCCTG AGTCAGAAGG GTATCACTGC CACCAAGCGG 294
AFR-23270 AGTAAAGAAT TTCGCGGCAG CCGTCCAGGA GATCAAGCCA CTCAATGAAC AGGTCCAC-- -ACTGCCTTG AGTCGGAAGG GTATCACCGC CACCCAGCGG 297
AFR-53993 AGTAAAGAAT TTCGCGGCAG CCGTCCAGGA GATCAAGCCA CTCAATGAAC AGGTCCAC-- -ACTGCCTTG AGTCGGAAGG GTATCACCGC CACCCAGCGG 297
ATH-A01 GATTAAGGAT TTTGCCCATG CTGTTGCGCA GATCAAACCC CTGAACGTGC AGGCTCAC-- -TCTGAAATC AGTCAGAAAG GCGTGAGTAA AGCCAAGCAG 216
ATH-LCY GATTAAGGAT TTTGCCCATG CTGTTGCGCA GATCAAACCC CTGAACGCGC AGGCTCAT-- -GCTGAAATC AGTCAGAAAG GCGTGAGTAA AGCCAAGCAG 216
ATH-19377 GATTAAGGAT TTTGCCCATG CTGTTGAGCA GATCAAACCC CTGAACGCGC AGGCTCAT-- -GCTGAAATC AGTCAGAAAG GCGTGAGTAA AGCCAAGCAG 216
ACA-SM1 GCTCCACGAT TTTGCCGGCG CCGTCAAGGA GTTGCAGGGT CTGAACAACC AGGTGCACCA GGAGGCCATG AATCCCAAGC ---TGACCCC GGCGCAAAAG 243
ACA-51756 GCTCCACGAT TTTGCCGGCG CCGTCAAAGA GTTGCAGGGT CTGAACAACC AGGTGCACCA GGAGGCCATG AATCCCAAGC ---TGACCCC GGCGCAAAAG 243
Consensus GATAAAGGAT TTTGCCGCCG CCGTTCAGGA GATCAAGCCA CTGAACGAAC AGGTCCAC-- -NCTGCCATG AGTCAGAAGG GTATGACTGC CGCCAAGCAG
Conservation
2.0bits
Sequence logo
0.0bits

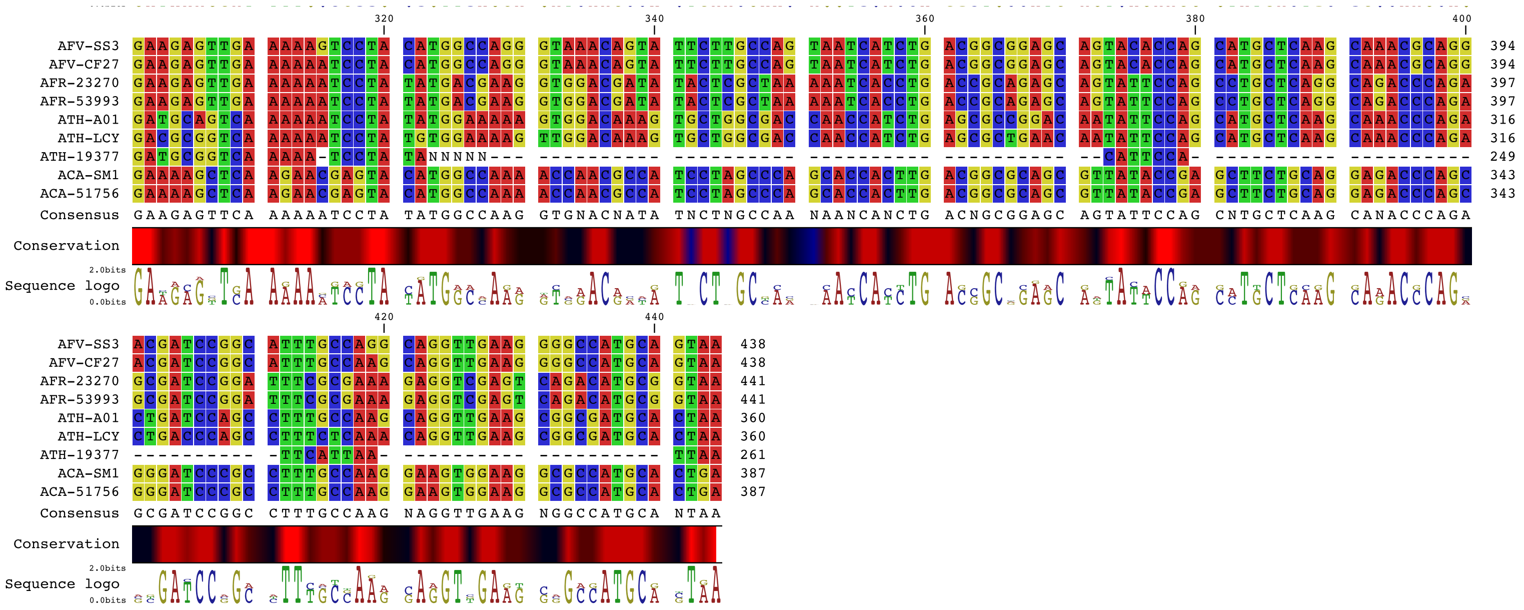

320 340 360 380 400
AFV-SS3 GAAGAGTTGA AAAAGTCCTA CATGGCCAGG GTAAACAGTA TTCTTGCCAG TAATCATCTG ACGGCGGAGC AGTACACCAG CATGCTCAAG CAAACGCAGG 394
AFV-CF27 GAAGAGTTGA AAAAATCCTA CATGGCCAGG GTAAACAGTA TTCTTGCCAG TAATCATCTG ACGGCGGAGC AGTACACCAG CATGCTCAAG CAAACGCAGG 394
AFR-23270 GAAGAGTTGA AAAAATCCTA TATGACGAAG GTGGACGATA TACTCGCTAA AAATCACCTG ACCGCAGAGC AGTATTCCAG CCTGCTCAGG CAGACCCAGA 397
AFR-53993 GAAGAGTTGA AAAAATCCTA TATGACGAAG GTGGACGATA TACTCGCTAA AAATCACCTG ACCGCAGAGC AGTATTCCAG CCTGCTCAGG CAGACCCAGA 397
ATH-A01 GATGCAGTCA AAAAATCCTA TATGGAAAAA GTGGACAAAG TGCTGGCGAC CAACCATCTG AGCGCCGGAC AATATTCCAG CATGCTCAAG CAAACCCAGA 316
ATH-LCY GACGCGGTCA AAAAATCCTA TGTGGAAAAG TTGGACAAAG TGCTGGCGAC CAACCATCTG AGCGCTGAAC AATATTCCAG CATGCTCAAG CAAACCCAGA 316
ATH-19377 GATGCGGTCA AAAA-TCCTA TANNNNN--- ---------- ---------- ---------- ---------- --CATTCCA- ---------- ---------- 249
ACA-SM1 GAAAAGCTCA AGAACGAGTA CATGGCCAAA ACCAACGCCA TCCTAGCCCA GCACCACTTG ACGGCGCAGC GTTATACCGA GCTTCTGCAG GAGACCCAGC 343
ACA-51756 GAAAAGCTCA AGAACGAGTA CATGGCCAAA ACCAACGCCA TCCTAGCCCA GCACCACTTG ACGGCGCAGC GTTATACCGA GCTTCTGCAG GAGACCCAGC 343
Consensus GAAGAGTTCA AAAAATCCTA TATGGCCAAG GTGNACNATA TNCTNGCCAA NAANCANCTG ACNGCGGAGC AGTATTCCAG CNTGCTCAAG CANACCCAGA
Conservation
2.0bits
Sequence logo
0.0bits
420 440
AFV-SS3 ACGATCCGGC ATTTGCCAGG CAGGTTGAAG GGGCCATGCA GTAA 438
AFV-CF27 ACGATCCGGC ATTTGCCAAG CAGGTTGAAG GGGCCATGCA GTAA 438
AFR-23270 GCGATCCGGA TTTCGCGAAA GAGGTCGAGT CAGACATGCG GTAA 441
AFR-53993 GCGATCCGGA TTTCGCGAAA GAGGTCGAGT CAGACATGCG GTAA 441
ATH-A01 CTGATCCAGC CTTTGCCAAG CAGGTTGAAG CGGCGATGCA CTAA 360
ATH-LCY CTGACCCAGC CTTTCTCAAA CAGGTTGAAG CGGCGATGCA CTAA 360
ATH-19377 ---------- -TTCATTAA- ---------- ---------- TTAA 261
ACA-SM1 GGGATCCCGC CTTTGCCAAG GAAGTGGAAG GCGCCATGCA CTGA 387
ACA-51756 GGGATCCCGC CTTTGCCAAG GAAGTGGAAG GCGCCATGCA CTGA 387
Consensus GCGATCCGGC CTTTGCCAAG NAGGTTGAAG NGGCCATGCA NTAA
Conservation
2.0bits
Sequence logo
0.0bits
