# Supplementary material for: Bioinformatic Analyses of Unique (Orphan) Core Genes of the Genus Acidithiobacillus: Functional Inferences and Use As Molecular Probes for Genomic and Metagenomic/Transcriptomic Interrogation
Source: Front Microbiol. 2016 Dec 27;7:2035. doi: 10.3389/fmicb.2016.02035 (PMC5186765; doi:10.3389/fmicb.2016.02035)

SUPPLEMENTAL FILE 2

Protein Secondary Structure Predictions for Families I-V

Bioinformatic Analyses of Unique (Orphan) Core Genes of the Genus *Acidithiobacillus*: Functional Inferences and Use As Molecular Probes for Genomic and Metagenomic/Transcriptomic Interrogation

Carolina González, Marcelo Lazcano, Jorge Valdés and David S. Holmes

frontiers in Microbiology

Family I

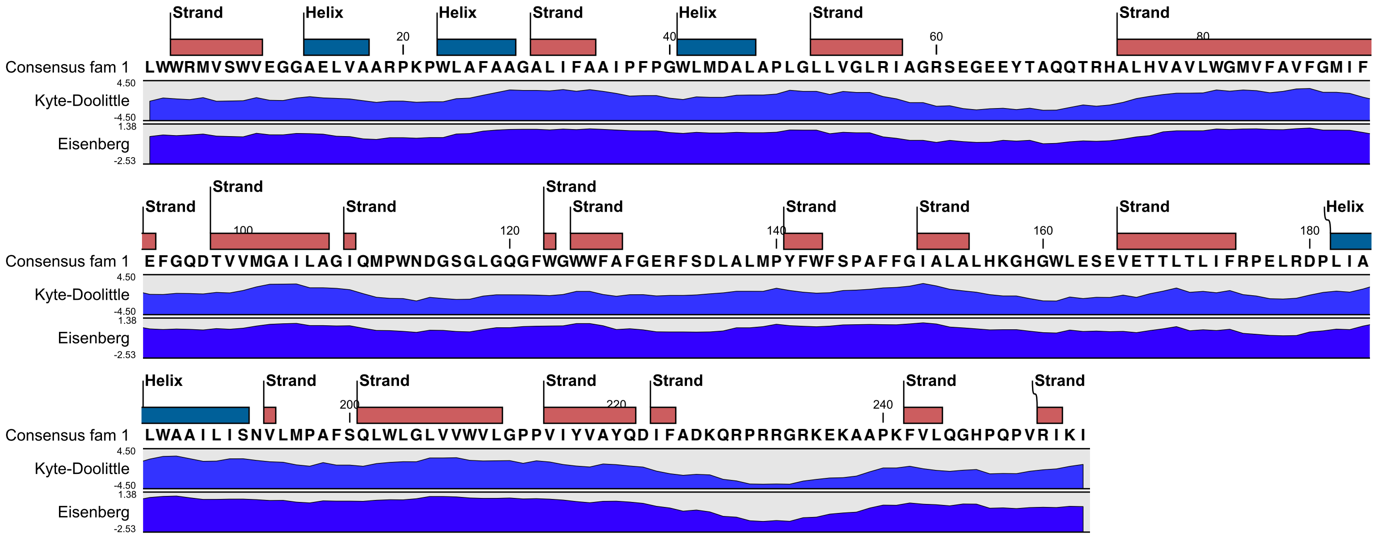

Family II

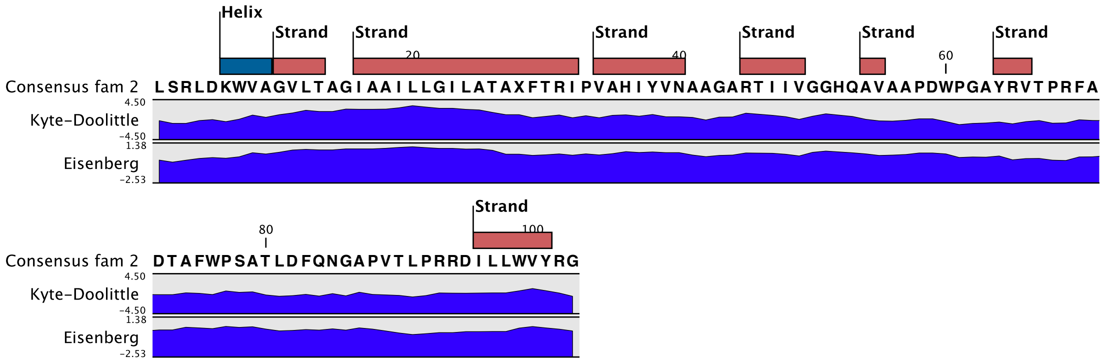

Family III

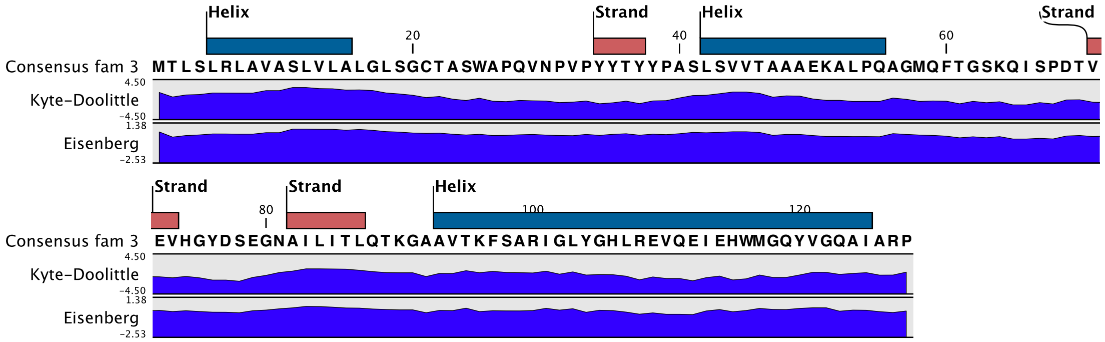

Family IV

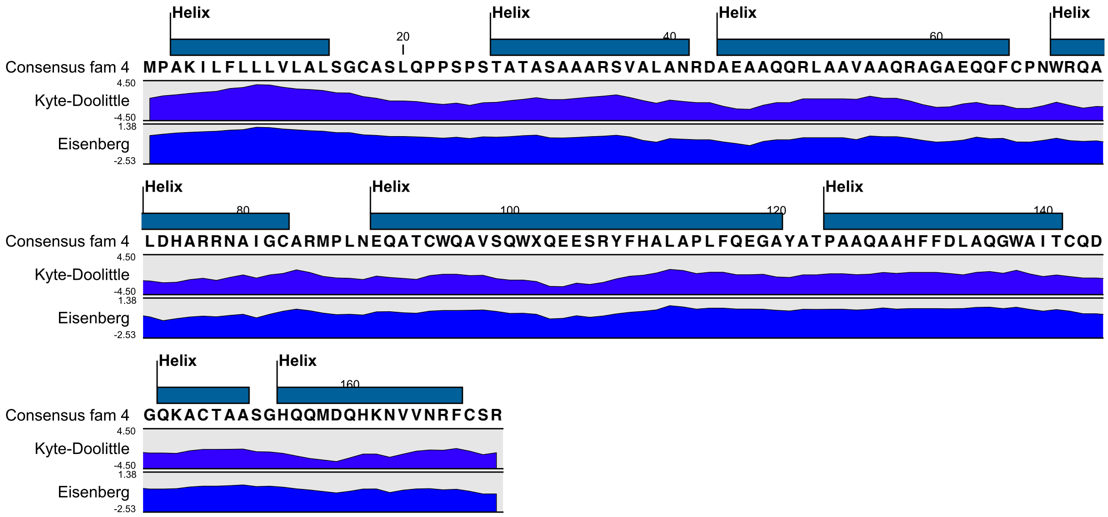

Family V

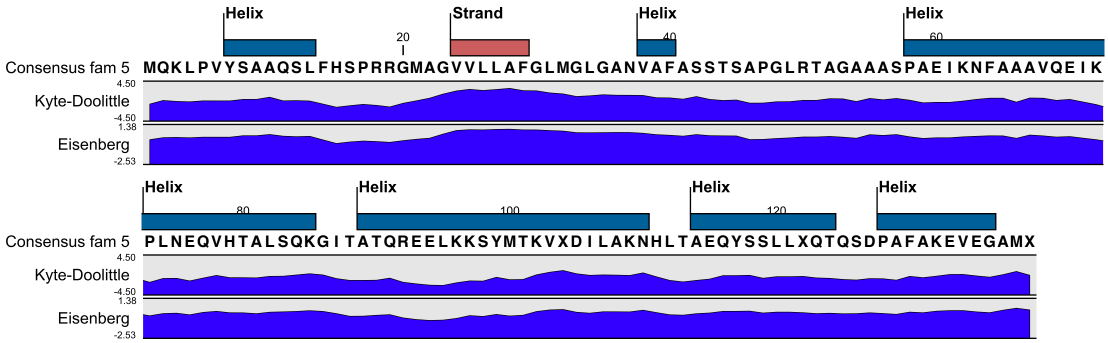

Supplement: Supplementary file 2 [file DataSheet2.pdf]
