# Supplementary material for: Bioinformatic Analyses of Unique (Orphan) Core Genes of the Genus Acidithiobacillus: Functional Inferences and Use As Molecular Probes for Genomic and Metagenomic/Transcriptomic Interrogation
Source: Front Microbiol. 2016 Dec 27;7:2035. doi: 10.3389/fmicb.2016.02035 (PMC5186765; doi:10.3389/fmicb.2016.02035)
Supplement: Supplementary file 3 [file DataSheet3.pdf]

SUPPLEMENTAL FILE 3

Genetic Context of Families I-V

Bioinformatic Analyses of Unique (Orphan) Core Genes of the Genus *Acidithiobacillus*: Functional Inferences and Use As Molecular Probes for Genomic and Metagenomic/Transcriptomic Interrogation

Carolina González, Marcelo Lazcano, Jorge Valdés and David S. Holmes

frontiers
in Microbiology

A.

FAMILY I

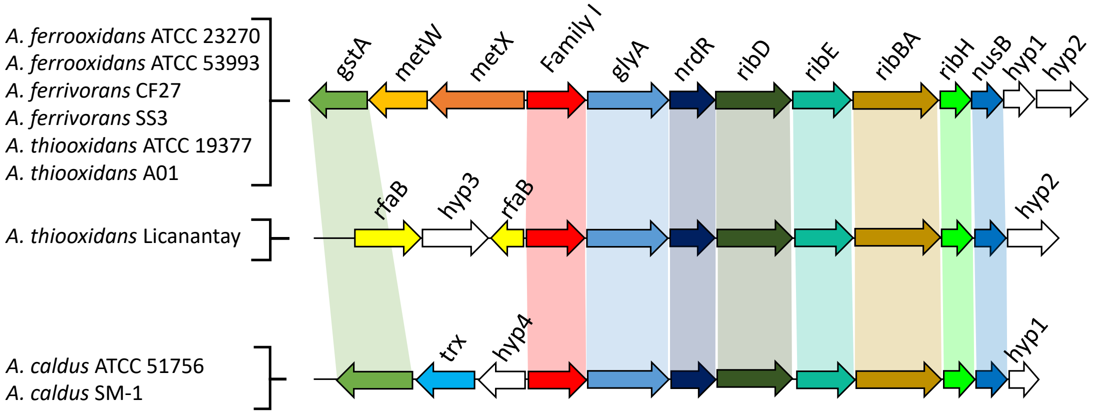

<u>Figure legend.</u>

gstA: Glutathione S-transferase.
metW: Methionine biosynthesis.
metX: Homoserine o-acetyltransferase.
Family I: Hypothetical.
glyA: Serine Hydroxymethyl transferase.
nrdR: Ribonucleotide reductase.
ribD: Riboflavin biosynthesis.
ribE: Alpha-Riboflavin biosynthesis.
ribBA: 3,4-dihydroxy-2 butanone 4-phosphate synthase.
ribH: 6,7-dimethyl-8-ribityllumazine synthase.
nusB: Transcription antitermination factor.
rfaB: Glycosyl transferase.
trx: Thioredoxin.
Hyp(1-4): Hypothetical protein (1-4).

B.

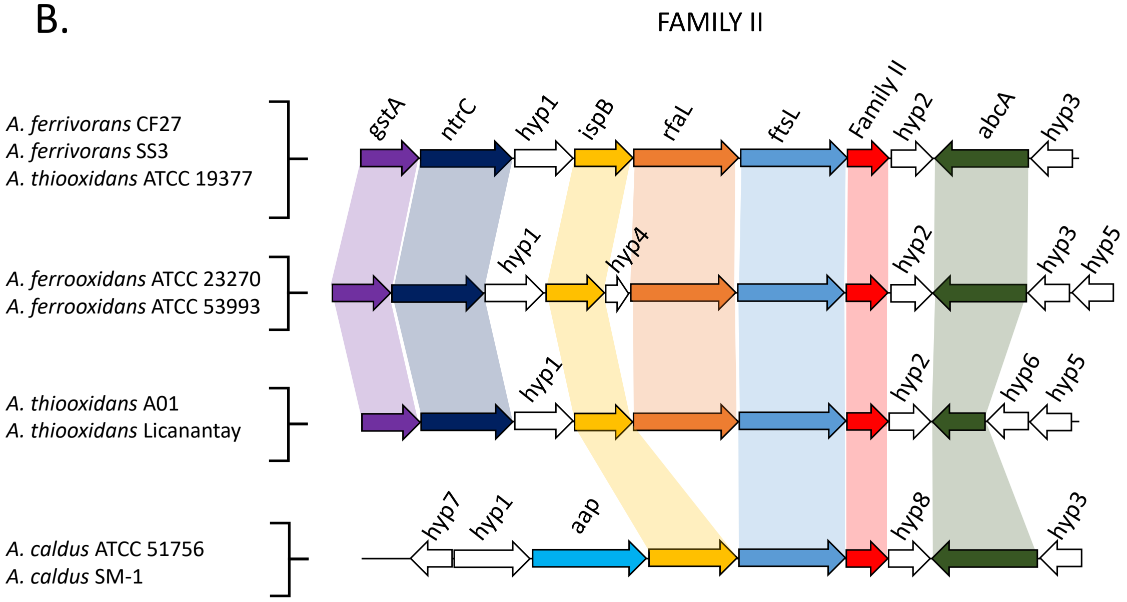

<u>Figure legend.</u>

gstA: Glutathione S-transferase.
ntrC: Nitrogen assimilation regulatory protein.
ispB: Octaprenyl-diphosphate synthase.
rfaL: O-antigen ligase.
ftsL: Cell division protein.
Family II: Hypothetical.
abcA: ABC transporter A family.
aap: Amino acid permease.
Hyp(1-8): Hypothetical protein (1-8).

C.

FAMILY III

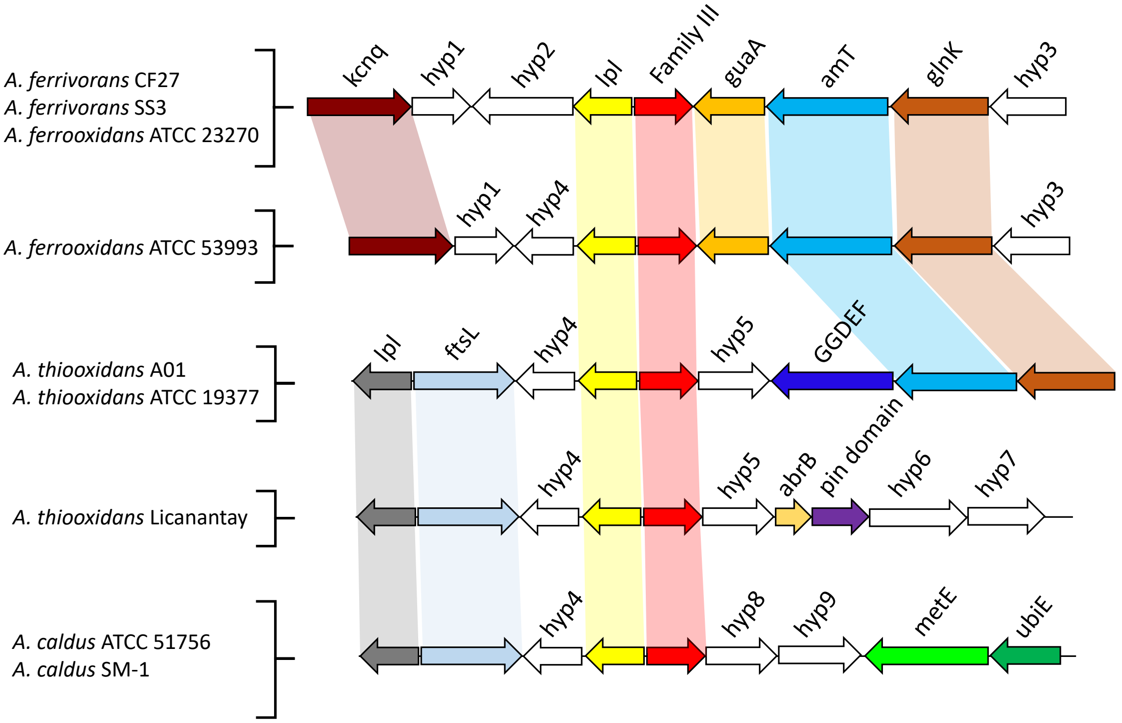

Figure legend.

kcnq: Potassium voltage-gated channel.
lpl: Lipoprotein.
Family III: Hypothetical.
guaA: Glutamine amino transferase.
amT: Ammonia transporter.
glnK: Nitrogen Regulatory.
ftsL: Cell division protein.
GGDEF: Diguanilate cyclase.
abrB: Transition state regulatory.
pin Domain: DNA binding protein.
metE: Methionine synthase.
ubiE: Ubiquinone biosynthesis.
Hyp(1-9): Hypothetical proteins (1-9).

D.

FAMILY IV

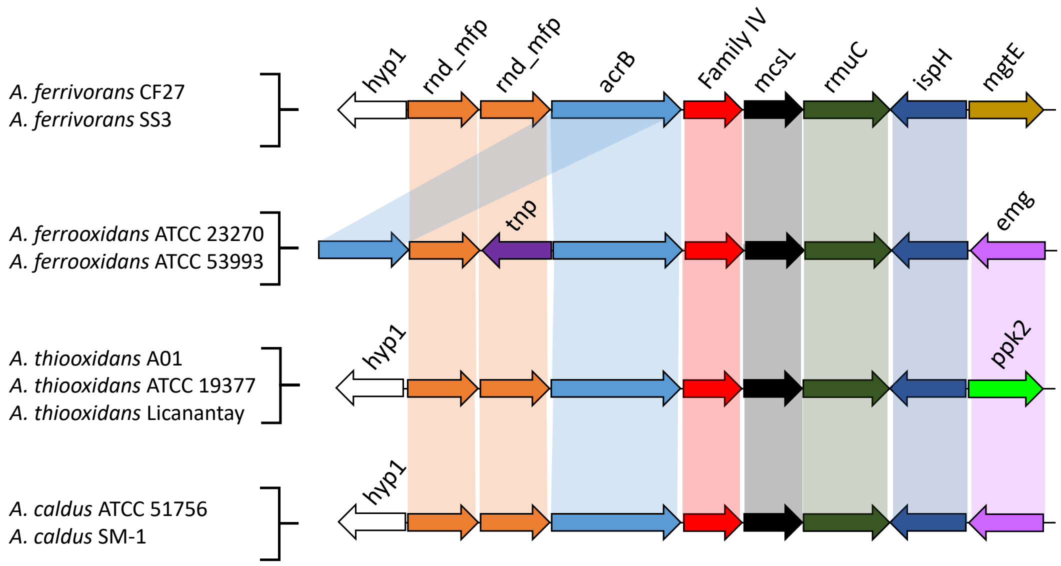

Figure legend.

rnd_mfp: RND transporter.
lpl: Lipoprotein.
acrB: Multidrug efflux RND transporter.
Family IV: Hypothetical.
mcsL: Large conductance mechanosensitive channel.
rmuC: DNA recombination protein.
ispH: 4-hydroxy-3-methylbut-2-enyl diphosphate reductase.
mgtE: Magnesium transporter.
tnp: Transposase.
emg: Ribosomal RNA small subunit methyltransferase.
ppk2: Polyphosphate kinase 2.
hyp1: Hypothetical protein 1.

E.

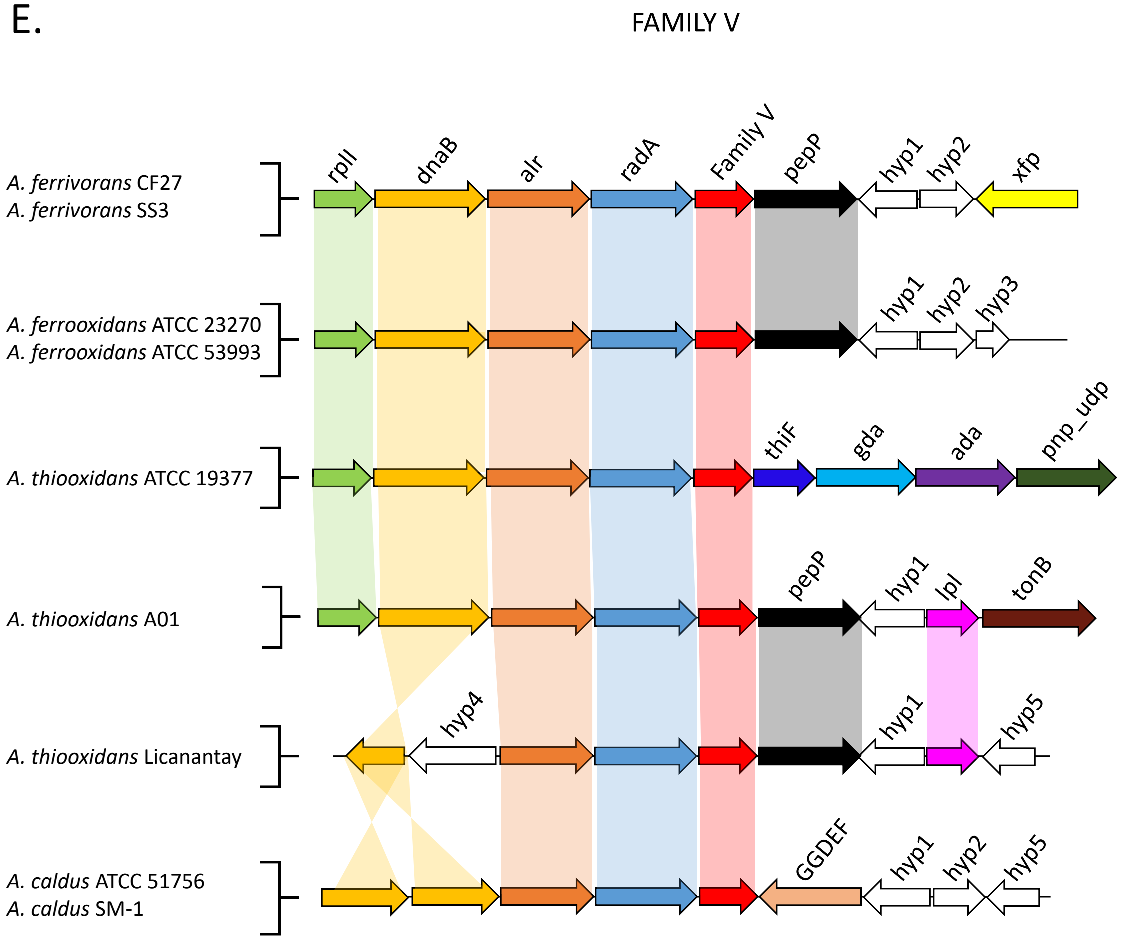

<u>Figure legend.</u>

rplI: Ribosomal 50 S protein.
dnaB: DNA helicase.
alr: Alanine racemase.
radA: DNA repair protein
Family V: Hypothetical.
pepP: Xaa-Pro aminopeptidase.
xfp: Phosphoketolase.
thiF: Thiazole biosynthesis adenylyltransferase.
gdA: Guanine deaminase.
adA: Adenosine deaminase.
pnp_udp: Phosphorylase.
lpl: Lipoprotein.
tonB: Transport protein.
GGDEF: Diguanilate cyclase
hyp(1-5): Hypothetical protein (1-5)
